# Discovery of the first ichthyosaur from the Jurassic of India: Implications for Gondwanan palaeobiogeography

**DOI:** 10.1371/journal.pone.0185851

**Published:** 2017-10-25

**Authors:** Guntupalli V. R. Prasad, Dhirendra K. Pandey, Matthias Alberti, Franz T. Fürsich, Mahesh G. Thakkar, Gaurav D. Chauhan

**Affiliations:** 1 Department of Geology, Centre for Advanced Studies, University of Delhi, Delhi, India; 2 Earth and Planetary Sciences Group, Manipal Center for Natural Sciences, Manipal University, Manipal, Karnataka, India; 3 Institut für Geowissenschaften, Christian-Albrechts-Universität zu Kiel, Kiel, Germany; 4 GeoZentrum Nordbayern, Friedrich-Alexander-Universität Erlangen-Nürnberg, Erlangen, Germany; 5 Department of Earth and Environmental Science, KSKV Kachchh University, Bhuj, India; Institute of Botany, CHINA

## Abstract

An articulated and partially preserved skeleton of an ichthyosaur was found in the Upper Jurassic (Upper Kimmeridgian) Katrol Formation exposed at a site south of the village Lodai in Kachchh district, Gujarat (western India). Here we present a detailed description and inferred taxonomic relationship of the specimen. The present study revealed that the articulated skeleton belongs to the family Ophthalmosauridae. The new discovery from India further improves the depauperate fossil record of ichthyosaurs from the former Gondwanan continents. Based on the preserved length of the axial skeleton and anterior part of the snout and taking into account the missing parts of the skull and postflexural region, it is suggested that the specimen may represent an adult possibly reaching a length of 5.0–5.5 m. The widespread occurrence of ophthalmosaurids in the Upper Jurassic deposits of western Tethys, Madagascar, South America and India points to possible faunal exchanges between the western Tethys and Gondwanan continents through a southern seaway.

## Introduction

Ichthyopterygia is a highly successful group of marine reptiles that appeared in the Early Triassic (Olenekian) from a possible diapsid ancestral lineage, survived until the middle Late Cretaceous (Late Cenomanian) and had a worldwide distribution [1, 2]. Though the diversity of ichthyosaurs suffered heavily during the end-Triassic mass extinction, they recovered in the earliest part of the Jurassic Period as evident from a large number of articulated and exceptionally preserved specimens reported from England, Germany and North America [3, 4]. However, their disparity had never recovered after the Triassic-Jurassic mass extinction [4]. Traditionally, all the Early Jurassic ichthyosaurs were considered to have been replaced during the Middle Jurassic by the ophthalmosaurids, a new clade of ichthyosaurs that appeared at the Aalenian-Bajocian boundary and survived into the Cretaceous [3, 5]. But the recent discovery of a basal thunnosaurian from Iraq demonstrated that non-ophthalmosaurid ichthyosaurs of Late Triassic radiation event survived into the Cretaceous [6]. In comparison to the Early Jurassic, the fossil record of Middle Jurassic ichthyosaurs is less known. The fossil record for the Late Jurassic is relatively better known and has been improving in recent years with many new discoveries coming from South America [5, 7–9], North America [10], and Europe [11–13].

Due to the extensive collections of ichthyosaurs from the UK and mainland Europe, historically the research on ichthyosaurs was biased towards the Laurasian continents. In the former Gondwanaland, the Jurassic ichthyosaur fossil record is very limited with most reports coming from the Middle and Upper Jurassic strata of Argentina [5, 7–9]. Until now, Jurassic ichthyosaurs have been reported from the Aalenian-Bajocian and Tithonian-Berriasian strata of Argentina [5, 7–9, 14–17], Tithonian of Chile [18], Tithonian of Madagascar [19], Rhaetian-Sinemurian of New Zealand [20], and Late Kimmeridgian-Middle Tithonian of Antarctica [21, 22]. Despite their widespread occurrence in the Jurassic, no ichthyosaur has been documented from the Jurassic of India until now.

In India, ichthyosaur remains have been reported previously from the Lower Cretaceous (Upper Albian—Middle Cenomanian) Karai Formation of the Cauvery Basin in South India [23–26]. But these finds are known primarily by a few isolated vertebrae and teeth. However, Lydekker [24] assigned a few complete and partially complete vertebrae to a new species *Ichthyosaurus indicus* because of a deep concavity of the centra. *I*. *indicus* was considered nomen dubium by McGowan and Motani [3]. Five complete adult and juvenile teeth from the same formation were assigned to *Platypterygius indicus* because of the morphological similarity of the teeth to *Platypterygius* and proximity of the two ichthyosaur fossil sites within the Karai Formation [25]. Zammit [27] observed that tooth forms similar to that of *Platyptrerygius* also occur in *Brachypterygius* and thus suggested that the placement of teeth from the Karai Formation in *P*. *indicus* is tentative. Later reviews of *Platypterygius* species have shown that the vertebrae reported by Lydekker are referable to Ichthyosauria indet. as they lack diagnostic characters. Further it was felt that among the teeth only DUGF/41 can be referred to subfamily Platypterygiinae gen. indet. because of its square rooted cross-section and the rest should be placed in the order Ichthyosauria fam. indet. [1, 28]. Although India hosts extensive marine Jurassic deposits both in the Himalayan and peninsular Indian (Kachchh, Jaisalmer) regions, until now no ichthyosaur remains have been documented from this time interval.

**Fig 1:**
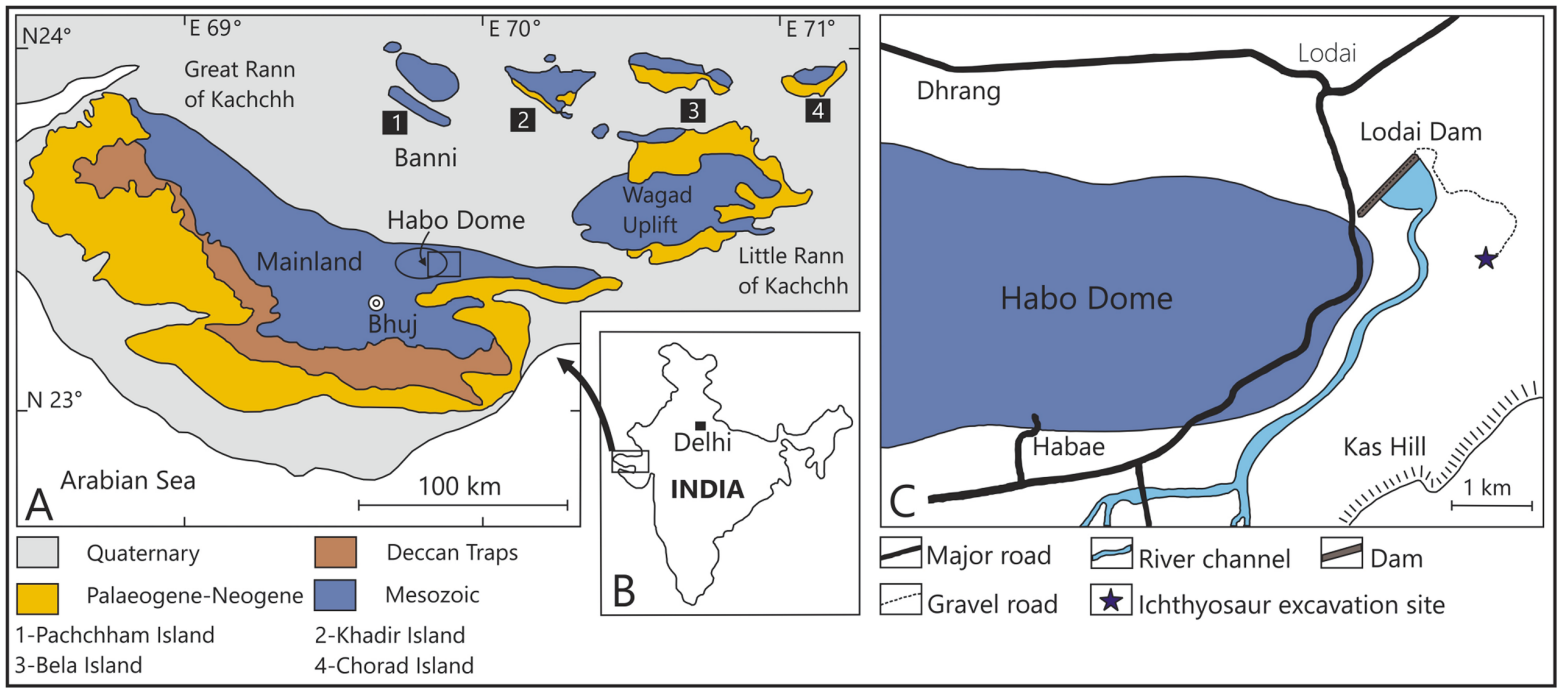
Geological and location map of the Upper Jurassic ichthyosaur site of Kachchh, western India. A. Geological map of the Kachchh region (modified after Fürsich et al. [39]), B. Inset map of India showing the location of Kachchh. C. Location map of the ichthyosaur site.

The Jurassic succession of the Kachchh Basin preserves rocks ranging in age from Aalenian to Tithonian but has yielded only a few marine reptiles. One plesiosaurian vertebra associated with a few fragmentary rib bones was documented from the Upper Tithonian to Neocomian Bhuj Formation from a site near Umia (Amiya) village and was assigned to *Thaumatosaurus indicus* Lydekker [29]. Bardet et al. [30] described 19 articulated vertebrae recovered from the lower part of the Katrol Formation corresponding to the *Katrolensis* Ammonite Zone, equivalent of the Beckeri Zone of the Western Tethys, as representing the plesiosaur family Cryptoclididae. They also redescribed a plesiosaur mandibular symphysis from the Lower Cretaceous Bhuj Formation, Kachchh, housed in the Indian Museum, Kolkata. This specimen, initially referred to *Thaumatosaurus indicus* [23, 31], was reassigned to *Simolestes indicus* [30]. Two large skulls of Callovian marine crocodiles putatively identified as *Steneosaurus* were also reported from marls of the Chari Formation underlying the Dhosa Oolite about 10 km southwest of Bhuj [32]. The relative scarcity of marine reptiles from the Jurassic deposits of India seems to be an artifact of sampling bias rather than of preservation potential of the fossils as these marine sequences have not been prospected in the past with a focused objective of recovering vertebrate fossils. During a field campaign carried out in January and February 2016, we discovered a partially preserved ichthyosaur skeleton (KGMV-0501) in the Upper Jurassic (Upper Kimmeridgian) Katrol Formation south of Lodai village (N 23° 22.391', E 69° 54.690'), about 30 km northeast of Bhuj in the Kachchh district, western India (Fig 1). KGMV-0501 represents the first Jurassic ichthyosaur and the first articulated ichthyosaur skeleton from the Mesozoic strata of India and is one of the few ichthyosaur finds from the former Gondwanaland. In this paper, a preliminary description of the newly discovered skeleton is presented and its dietary adaptations as well as palaeobiogeographic significance are discussed.

## Geological setting and age

The Kachchh Basin is a pericratonic E-W oriented rift basin on the western margin of India, which was formed during Late Triassic rifting between India and Africa [33]. In the Kachchh region, Jurassic rocks are exposed in three areas, the Kachchh Mainland, the Island Belt (Pachchham, Khadir, Bela and Chorad) within the salt marshes of the Great Rann of Kachchh, and the Wagad Uplift in its eastern part (Fig 1).

The Mesozoic rocks of Kachchh were first described by Wynne [34]. However, it was Waagen [35] who divided these rocks into four subdivisions viz., Patcham, Chari, Katrol and Umia Series in ascending order. Subsequently, many revised classifications have been proposed for the Jurassic stratigraphy of Kachchh but the names proposed by Waagen [35] survived as formal lithostratigraphic units until now. According to Fürsich et al. [36], the Jurassic sequence of Kachchh commences with an initial phase of continental sedimentation of Late Triassic to Early Jurassic age. Following this phase of terrestrial sedimentation, marine depositional environments prevailed in the basin from the Bajocian to Aptian with a hiatus in sedimentation from the upper part of the Oxfordian to the lower part of Kimmeridgian [37, 38]. The Kimmeridgian in the Kachchh basin is represented by a thick succession of siliciclastics belonging to the Katrol Formation. In the early Middle to Late Kimmeridgian, shallow-marine to mid shelf conditions prevailed throughout the basin [39].

**Fig 2:**
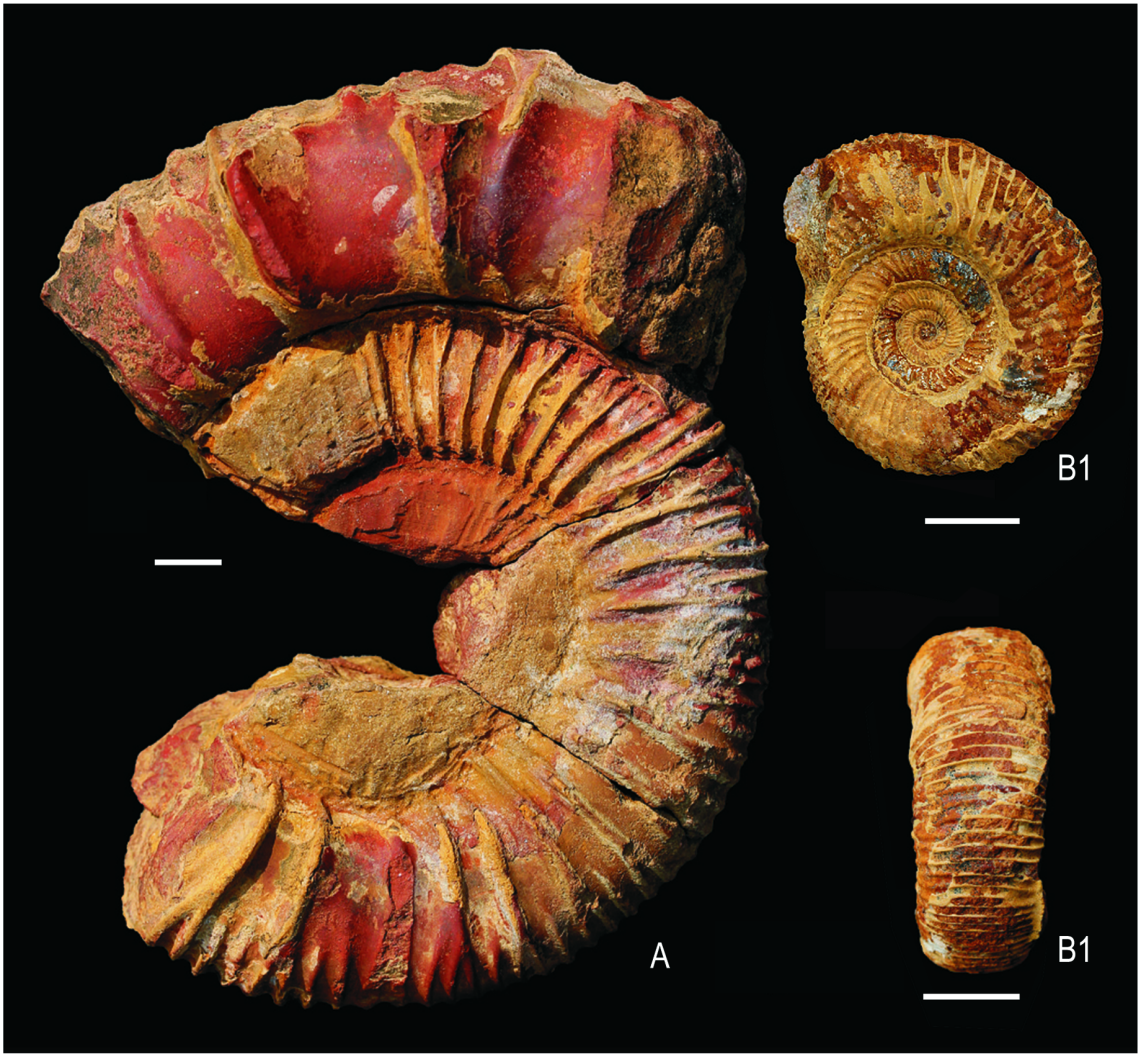
*Katroliceras lerense* Spath, 1931 (RUC2016IKH 01) in lateral view (A). Inner whorl of *Katroliceras* sp. (RUC2016IKH 02) (B1-B2) from the lower Katrol Formation (Upper Kimmeridgian) at the study site in lateral (B1) and ventral (B2) views. Scale bars equal 1 cm.

The basal sandstones of the Katrol Formation on the Kachchh Mainland yielded ammonites of late Early to Late Kimmeridgian age at a number of localities. The present ichthyosaur has been found within the lower Katrol Formation together with a number of ammonites belonging to *Katroliceras lerense* Spath, 1931 and *Katroliceras* sp. (Fig 2). This index fossil indicates the Katrolensis Zone of the Upper Kimmeridgian [40], which corresponds to the Beckeri Zone of the north Tethyan margin [41].

## Material and methods

Field investigations carried out by a team comprising the present authors and students of Delhi University and KSKV Kachchh University during the months of January and February 2016 resulted in the discovery of a fossil vertebrate yielding horizon in the Upper Kimmeridgian Katrol Formation to the south of Lodai village near Bhuj town, Gujarat, India (Fig 1). During a full scale excavation at this fossil site, the vertebral column, ribs, neural spines, gastralia and two associated fins were found in articulation. Additionally, a part of the snout representing the premaxilla was found at the anterior end of the preserved vertebral column and a few isolated teeth, vertebrae, jaw fragments, and other bone fragments were found scattered around the excavation site. The skeleton is encased in a hard, ferruginous nodular matrix whereas the ribs are preserved in a soft shale. The vertebral column preserved a portion of the cervical region, the entire dorsal region, and a part of the pre-flexural region and measures 3.6 m. Taking into consideration of 36 cm long preserved premaxillary bone, missing skull region and the postflexural vertebrae, it is inferred that the complete skeleton may have measured between 5.0 and 5.5 m in length. The axial skeleton and two paddles were collected in six plaster jackets. Before making the plaster jackets, photographs of the axial skeleton and associated paddles were taken using a Canon EOS 60D Camera. Also, the fragmented ribs were collected separately after taking photographs and making rough sketches. Line drawings of the two inferred forefins, isolated vertebrae, and the vertebral column were made from photographs. The specimens described here are deposited in the Geological Museum of the Department of Earth and Environmental Science, Krantiguru Shyamji Krishna Verma Kachchh University, Bhuj, Kachchh District, Gujarat, India and bear accession numbers KGMV 0501–0512. The present work was an integral part of a completed Government of India (Science and Engineering Research Board, New Delhi) funded research grant (SR/S2//JCB-14/2010) to GVRP.

### Mode of preservation

KGMV 0501 is found in a horizontal position on the bedding plane of greenish-yellow silty shales. The skeleton was lying on its left lateral surface exposing its right lateral side. One of the fins, inferred as the left forefin, was lying close to the anterior part of the vertebral column in its natural position. However, no bones of the pectoral girdle were found, but they may have been buried in the rock matrix underneath the ribs and vertebrae. A second fin of nearly the same size as the left forefin is found close to the tail bend. As the hindfins are highly reduced in post-Middle Jurassic ichthyosaurs and based on the size similarity of this fin to that of the anterior fin it is regarded here as most likely the right forefin. No bones of the girdle were found attached to the fin. This fin appears to have been displaced from its natural position before the axial skeleton rested on the sea floor as its proximal end is ventrally pointing with respect to the axial skeleton, whereas its distal end with fin elements is partially covered under the vertebral column. Some of the distal fin elements can be seen protruding dorsally above the vertebrae. Though no skull was found, an anterior part of the snout representing the premaxilla was found in a vertical position at the anterior end of the vertebral column. The skull may be present in one of the plaster jackets, but preparation is required. Articulated, highly fragmented ribs and gastralia lie on the ventral side of the vertebral column. The postfexural vertebrae are not preserved.

From the vertically positioned anterior part of the premaxilla, it is inferred that the animal after death nose-dived into the soft sediment with the skull going down into the substrate in a vertical position. Once the skull landed vertically in the substratum, the body was laid on its lateral side. The teeth in the premaxilla were dislodged from their natural position after death. Differential preservation of the skeleton with a major part remaining intact (anterior premaxilla, vertebral column and left forefin), one displaced possible right forefin, and non-preservation of hindfins, pectoral and pelvic girdles and part of the posterior tail bones may be interpreted in terms of scavenging, reworking, and partial burial of the skeleton with the exposed parts being subjected to disarticulation and decomposition [42]. The vertebral column is embedded in maroon-coloured ferruginous concretions. The nearly intact vertebral column indicates that the concretionary ferruginous coating of the skeleton took place following its burial below the sediment and this happened at least a few centimeters below the sediment-water interface. Possibly this prevented the disarticulation of the skeleton by any scavenger or by epifaunal activity. During its excavation ammonoid shells and belemnite rostra were found within the greenish-yellow shales of the Katrol Formation. The presence of ammonoids and belemnites indicate that the sediments were deposited under fully marine conditions.

## Systematic Palaeontology

Superorder Ichthyopterygia Owen, 1840

Order Ichthyosauria de Blainville, 1835

Family Ophthalmosauridae Baur, 1887

Ophthalmosauridae gen. et sp. indet.

(Figs 3–12)

### Referred material

Partially preserved skeleton (KGMV 0501) consisting of premaxilla, articulated vertebrae, left forefin, and? right forefin. Two fairly well-preserved teeth (KGMV 0502–0503), one posterior caudal vertebra (KGMV 0504), three postflexural caudal vertebrae (KGMV 0505–0507), three isolated phalanges (KGMV 0508–0510) and two mandibular fragments (KGMV 0511–0512) found scattered around the articulated skeleton are considered to belong to KGMV 0501.

### Stratigraphic horizon, age and locality

Greenish-yellow shales of the Katrol Formation representing the Katrolensis Zone of the Upper Kimmeridgian age [40] exposed near Lodai village (N23°22.391': E69°54.690'), 30 km northeast of Bhuj, Kachchh District, Gujarat (western India).

### Description

**Fig 3:**
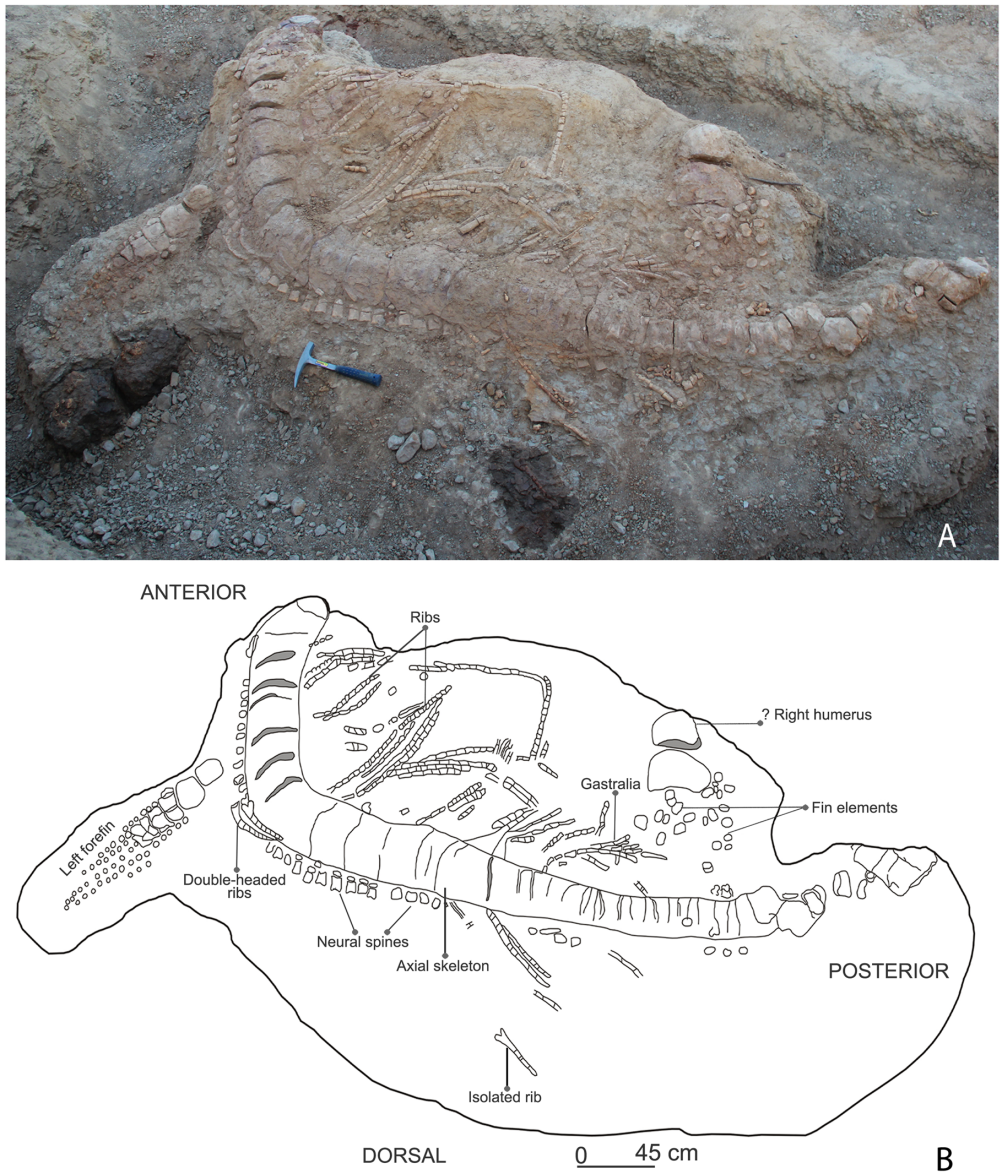
Field photograph (A) accompanied with a sketch (B) of the excavated ichthyosaur skeleton in the Katrol Formation near Lodai village, Kachchh, India.

The studied specimen comprises a large, partial skeleton that is mostly articulated (KGMV 0501). The preserved skeletal remains include an incomplete premaxilla, articulated vertebrae of cervical, dorsal and pre-flexural regions, left forefin, possible right forefin all in articulation and isolated teeth, vertebrae of posterior caudal and postflexural regions, and phalanges possibly belonging to the articulated skeleton. Some of the anterior vertebrae, many of the caudal vertebrae and perhaps the pelvic remains are missing. The fin lying close to the vertebral column anteriorly is the left forefin in its original position with only a slight dislocation from the axial skeleton (Fig 3). The posteriorly located fin, lying close to the tail just near the posterior ribs, possibly represents the right forefin. Despite its position, the latter is regarded as a displaced right forefin because hindfins became highly reduced in Jurassic and Cretaceous ichthyosaurs [43].

#### Premaxilla

**Fig 4:**
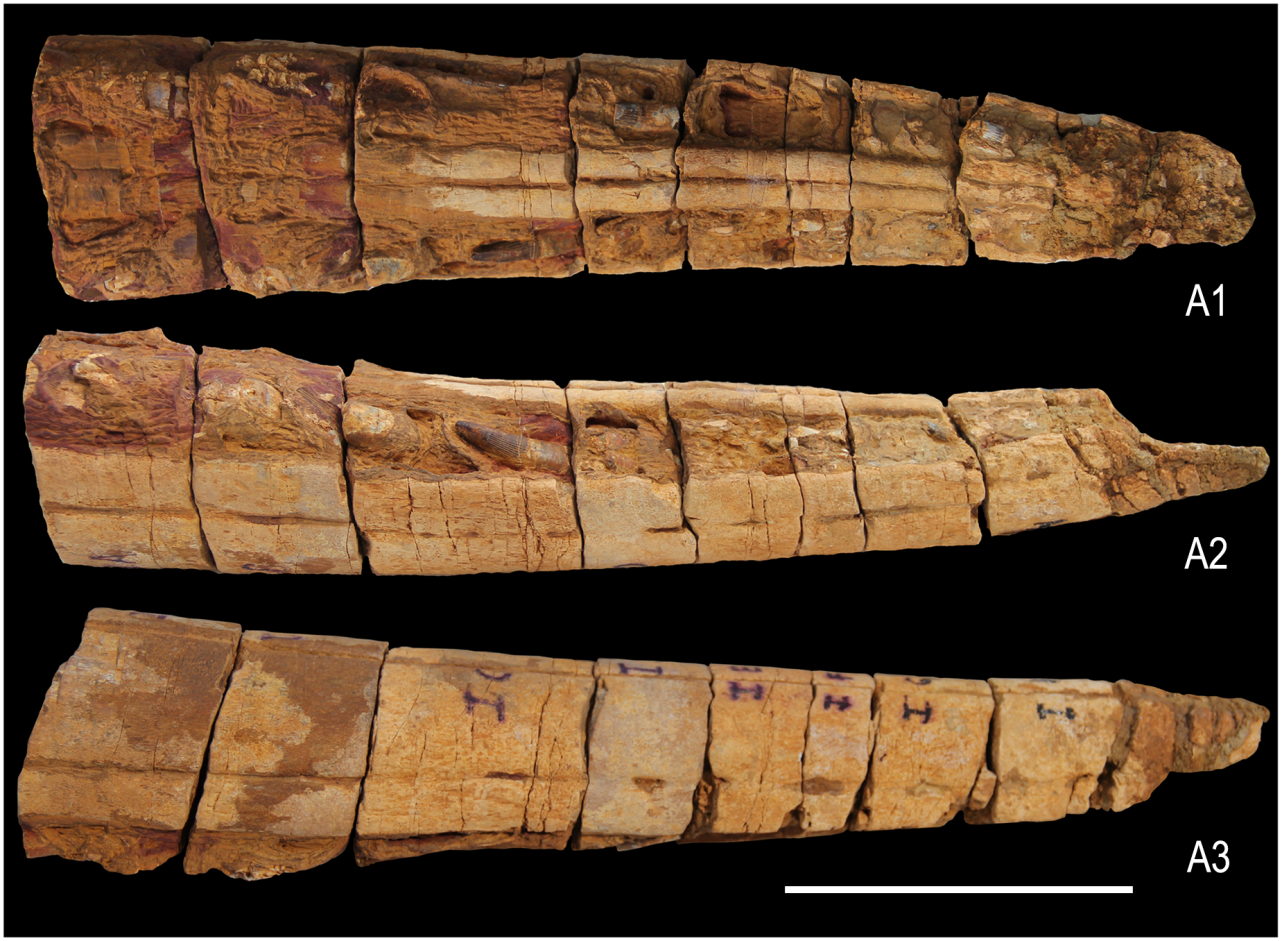
Anterior part of the premaxilla of Ophthalmosauridae gen. et sp. indet. (KGMV 0501) in ventral (A1), left lateral (A2) and right lateral (A3) views. Scale bar equals 10 cm.

**Fig 5:**
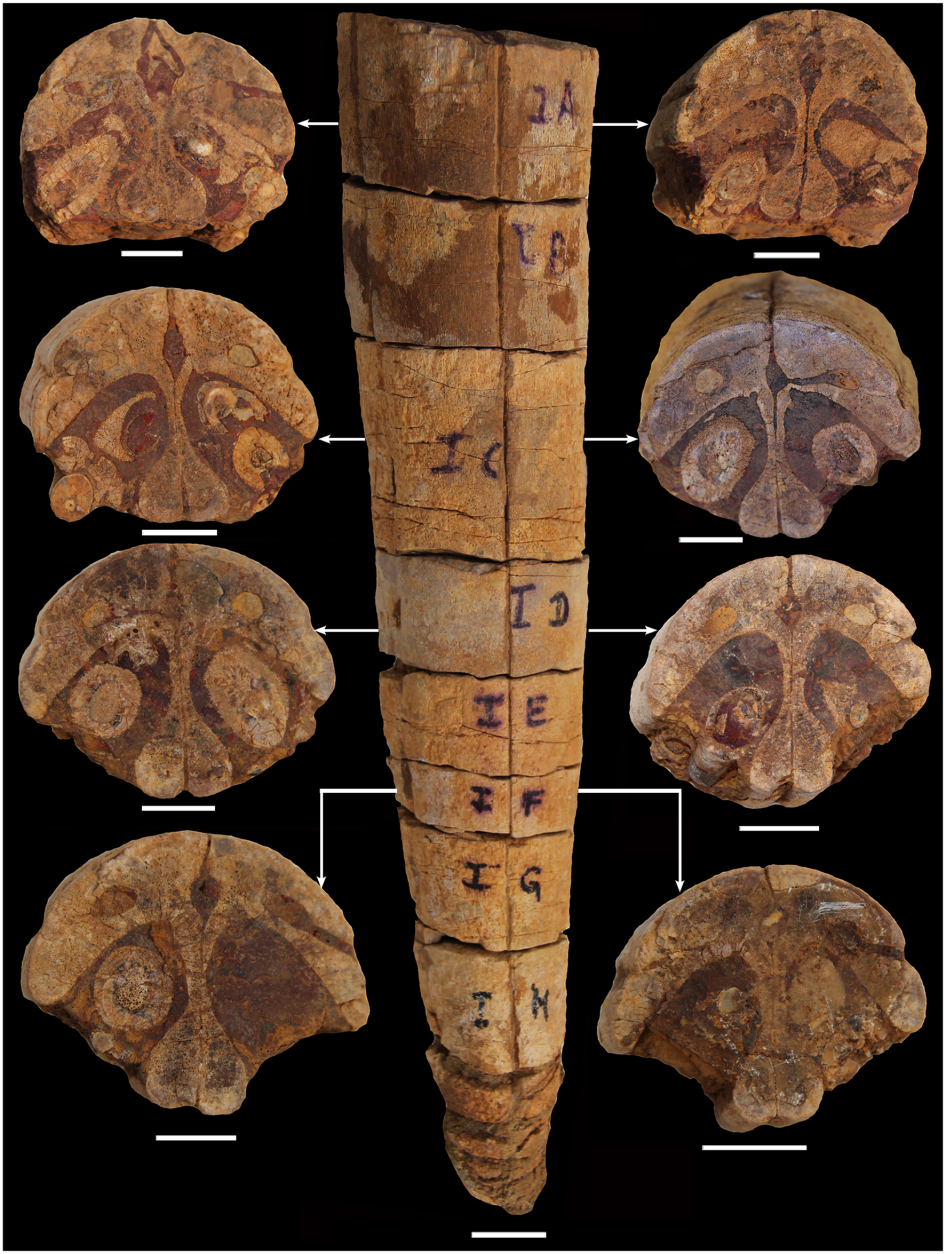
Anterior part of the premaxilla of Ophthalmosauridae gen. et sp. indet. (KGMV 0501) showing cross sections at posterior (left) and anterior (right) ends of the premaxilla at different segments of the bone. Scale bars equal 2.2 cm for the middle picture and 2 cm for the left and right pictures.

The incomplete premaxillary bone is 36 cm in length (Fig 4A1–4A3). The preserved bone represents the part of the premaxilla anterior to the nasal and up to the tip of the snout. It has a rounded dorsal surface and semi-circular cross-section (Fig 5). Both the right and left labial faces of the premaxilla bear a longitudinal groove (fossa premaxillaris) with a few nutrient foramina opening into its floor discretely and coalescing with it posteriorly (Fig 4A2–4A3). This fossa lies about 1.5 cm above the dental groove. The premaxilla is a semi-cylindrical bone each side having an outer facial and inner palatal part. The naturally sectioned premaxilla (8 fragments) offers 16 transverse sections for study (Fig 5). In transverse section, the facial part of the bone is semi-lunate in shape with a convex outer face and concave inner face (Fig 5). The facial pair of bones meets along the midline and forms the arch of the snout. Together with the outward diverging curved ends of the palatal bones, the semi-lunate facial bones enclose a lozenge-shaped canal in the midline that persists from the preserved distal part to the anterior end of the premaxilla. This canal, interpreted as a direct continuation of the cranial cavity [44], decreases in size anteriorly. In all preserved natural transverse sections, the two bones of the palatal process are not separated from the facial part. The palatal process occurs in the form of two curved vertical plates that enlarge at their ventral extremities into rounded, club-like structures (Fig 5). In the ventral face, the lower ends of the palatal process are rounded, closely approximated in the middle.

The broad groove between the palatal process and the facial part on either side is broad, deep and holds one tooth each (in cross section) (Figs 4A1–4A2 and 5). The teeth are held loosely in this dentigerous groove, which is oriented more vertically in the anterior part and becomes laterally oriented posteriorly. The exposed ventral surface of the premaxilla displays at least eight teeth in each half of the bone. But all the teeth are only partially preserved, small in size and oriented anteroposteriorly with their pointed ends slanting posteroventrally. In most cases, their tips are broken. This may be an artifact of post-mortem dislocation of the teeth from their vertical positions. In one transverse section, cross-sections of replacement teeth dorsal to the mature teeth have also been noticed (Fig 5). The posterior teeth are oriented slightly more laterally than the anterior ones. The state of preservation indicates that the teeth were lost or broken prior to burial.

#### Teeth

**Fig 6:**
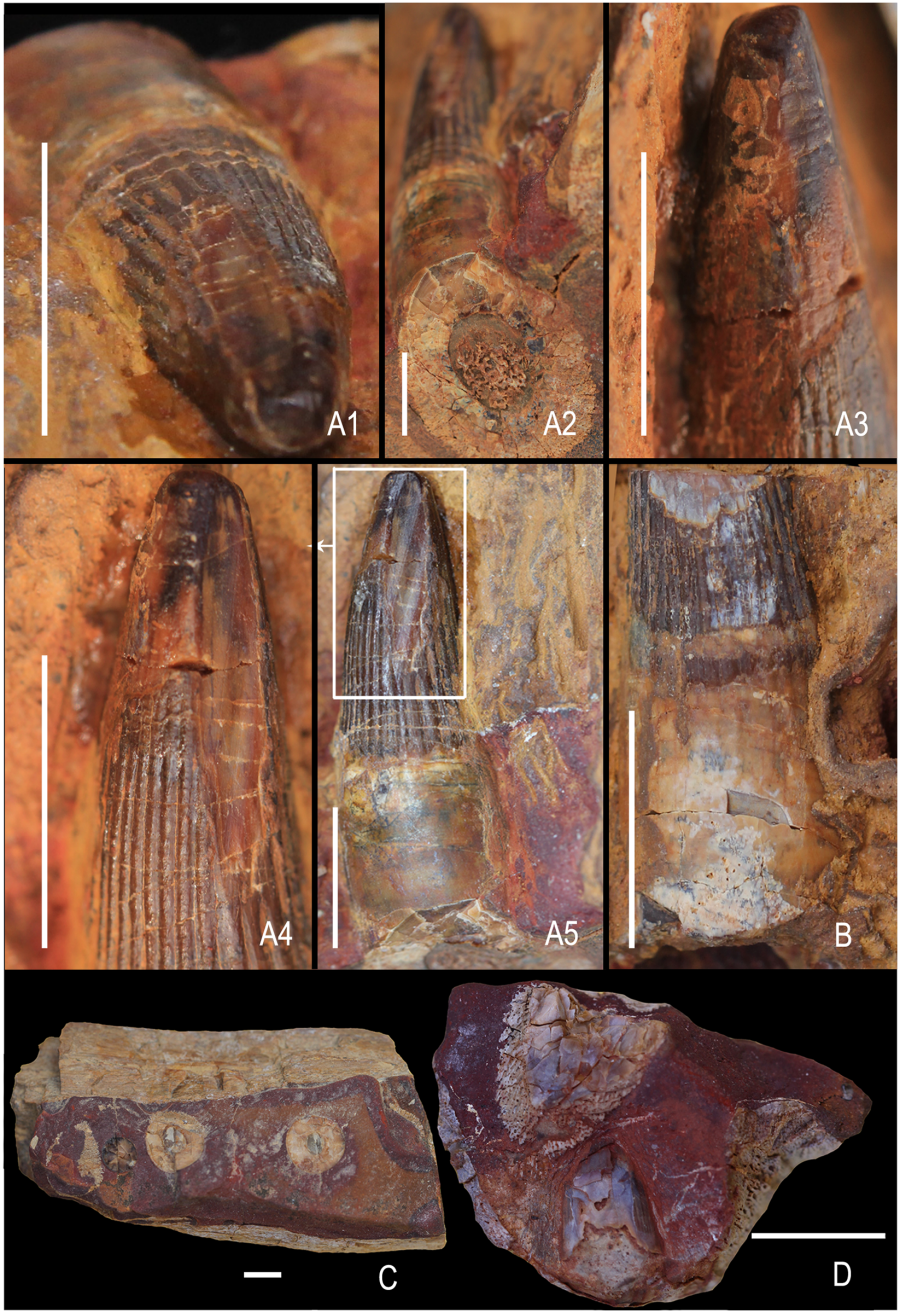
Ophthalmosauridae gen. et sp. indet. A1-A5, premaxillary tooth showing wear facet in apical view (A1), basal cross-section (A2), enamel spalling in the apicolabial region (A3), longitudinal enamel spalling on the linguolateral facet (A4), obliquely oriented grooves on the longitudinally spalled enamel (A5). A second premaxillary tooth showing breakage in the apical region (B), a mandibular fragment showing circular cross-section of teeth (KGMV 0511) (C), a small fragment of jaw showing an erupting tooth beneath an aged tooth (KGMV 0512) (D). Scale bars equal 1 cm.

One of the preserved teeth in the premaxilla is very robust (preserved height = 34 mm, maximum basal diameter = 17 mm) and has a worn rounded apex (Fig 6A1). This tooth has a slight lingually curved crown, a short and smooth acellular cementum layer and an oval cross-section. It shows enamel spalling on its labial face close to the apex (Fig 6A3) and extensive longitudinal enamel spalling on its lingual face that extends from the apex to nearly its base (Fig 6A4). The lingual longitudinal enamel spalling also exhibits obliquely oriented coarse grooves (Fig 6A5). The longitudinal fluting of this tooth is relatively coarse. Though not many teeth are preserved in the premaxilla, they appear to decrease in size towards the front. Most of the premaxillary teeth have broken tips (Fig 6B). However, it is unclear if the damage is due to post-mortem taphonomic processes or related to food processing. In one of the mandibular fragments (KGMV 0511), the broken teeth show a circular cross-section (Fig 6C).

**Fig 7:**
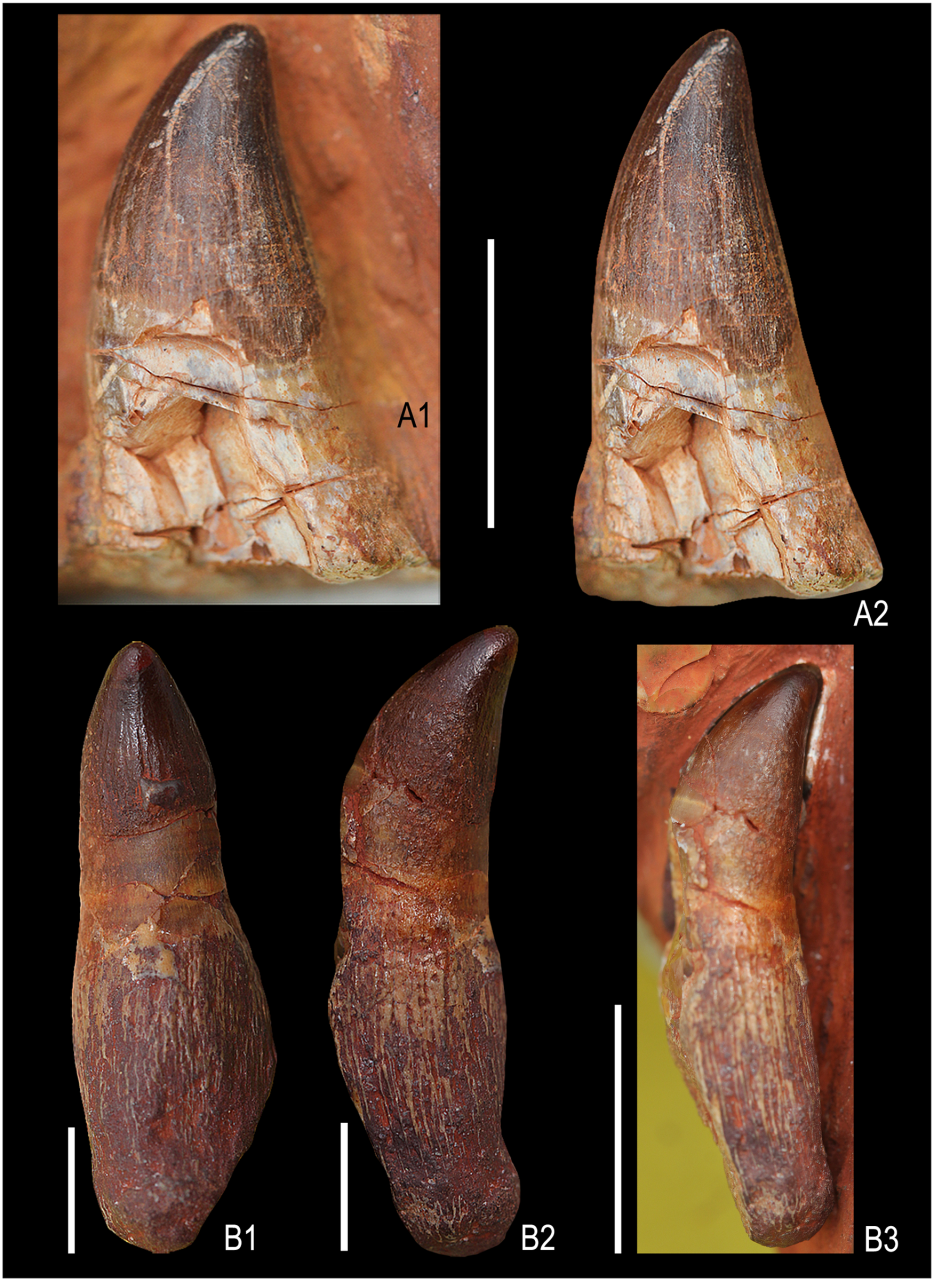
Isolated teeth of Ophthalmosauridae gen. et sp. indet. Lateral view of a tooth embedded in ferruginous matrix (KGMV 0502) (A1), the same tooth with edges improved by photoshop (A2). A second tooth (KGMV 0503) in lingual view (B1), lateral view (B2), embedded in ferruginous matrix before extraction (B3). Scale bars equal 1 cm.

Two teeth (KGMV 0502–0503) were found on the surface at the excavation site close to the anterior end of the articulated axial skeleton and probably belong to this specimen. KGMV 0503, the better preserved of the two teeth, has a crown with longitudinal striations that do not reach the apex, smooth and short acellular cementum (dentine) layer, and the basal elongated and flaring, porous cellular cementum with fine longitudinal ridges (Fig 7B1–7B3). In this tooth, the basal cellular cementum layer is nearly half of the total height of the tooth (crown height = 9 mm, height of acellular cementum = 7 mm, height of cellular cementum = 15 mm). The apex of the crown is smoothened by wear. Though KGMV 0503 has a long root with basal longitudinal striations, the shape of the basal cross-section cannot be deciphered, as the root is longitudinally broken (Fig 7B1–7B3). As this tooth has no strongly expanded root base as in *Platypterygius*, its cross-section may have been circular or oval in shape. KGMV 0502 is similar to KGMV 0503 in the morphology of its crown and smooth dentine layer, but its basal cellular cementum layer is not preserved (Fig 7A1 and 7A2). At the junction of the crown and acellular cementum, the tooth is inflated and the dentine-covered area is flaring ventrally and slightly compressed transversely rendering an elliptical basal cross section. In KGMV 0502, a longitudinal scratch mark extending from the tip to the middle of the crown possibly represents a wear surface (abrasion).

#### Axial skeleton

The preserved length of the axial skeleton is 3.6 m (Fig 3). The vertebral column is embedded in a hard ferruginous matrix and as a result no diagnostic characters to clearly differentiate the cervical vertebrae from dorsal or caudal vertebrae are observed. The description of the vertebral column is, therefore, not possible until the specimen is prepared. However, short and wide neural spines can be seen dorsally in the anterior and middle parts of the preserved axial skeleton (Fig 3). Since the preserved axial skeleton includes the region just behind the skull and extends up to the tail bend, it is anticipated that several cervical, all the dorsal and most of the pre-flexural vertebrae are present.

One large and three small vertebrae were collected as float during the excavation of the articulated skeleton. Based on the criteria used by McGowan and Motani [3] and Kirton [45], the isolated vertebrae from the Lodai ichthyosaur site can be identified as posterior caudal and postflexural vertebrae.

**Fig 8:**
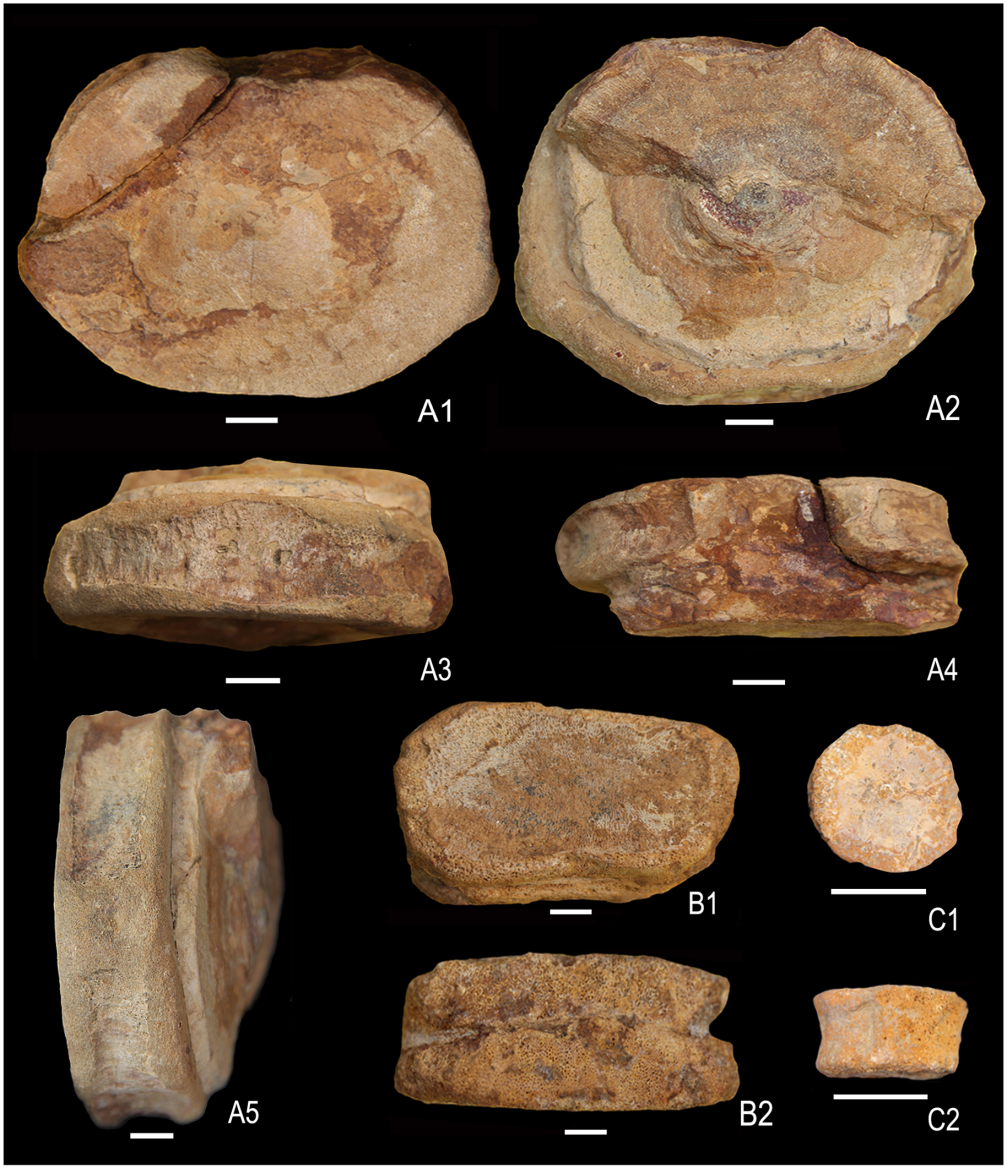
Ophthalmosauridae gen. et sp. indet. A1-A5 posterior caudal vertebra (KGMV 0504) in posterior (A1), anterior (A2), ventral (A3), dorsal (A4) and lateral views (A5) showing rounded facet for a rib. B1-B2. isolated proximal phalanx (KGMV 0508) in dorsal or ventral (B1) and lateral (B2) views. C1-C2. isolated distal phalanx (KGMV 0509) in dorsal or ventral (C1) and lateral (C2) views. Scale bars equal 1 cm.

**Fig 9:**
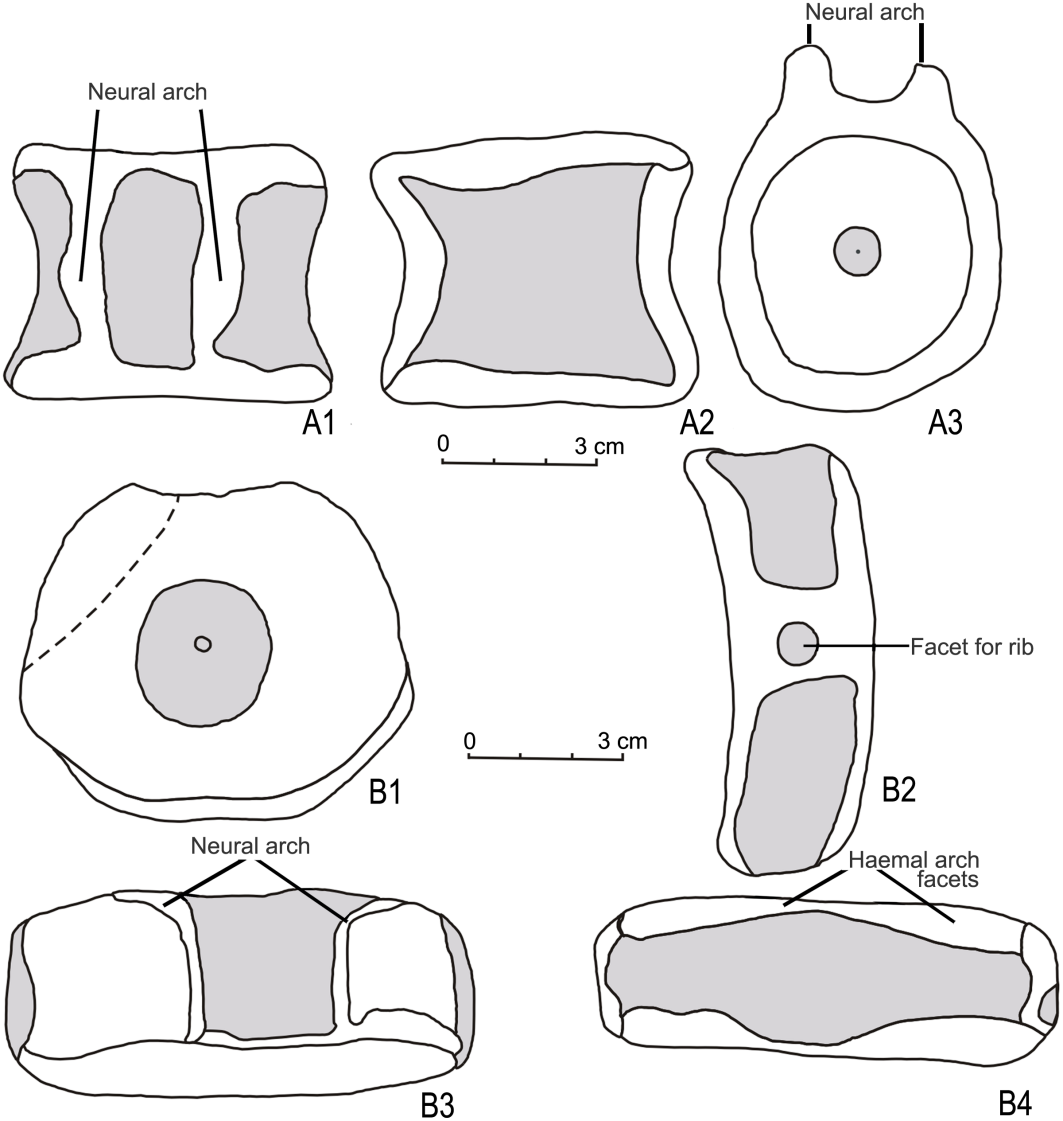
Ophthalmosauridae gen. et sp. indet. Line drawings of post-flexural caudal vertebra (KGMV 0505) (A1-A3) in dorsal (A1), lateral (A2), and anterior or posterior (A3) views. Line drawings of posterior caudal vertebra (KGMV 0504) (B1-B4) in posterior (B1), lateral (B2), dorsal (B3) and ventral (B4) views.

One specimen (KGMV 0504) of the present collection of isolated vertebral centra can confidently be identified with posterior caudal vertebra. KGMV 0504 is anteroposteriorly flattened and disc-like (length of the centrum = 2.7 cm) (Figs 8A1–8A5 and 9B1–9B4). In the anterior and posterior views, the centrum is sub-quadrangular in outline with a slightly broader ventral side (at the level of rib facet) (8.7 cm) than the dorsal side (8 cm). The height of the centrum at the level of neural arches is 7.8 cm. The rib facet is a broad and elevated ridge that extends over the entire length of the centrum slightly below its mid-height and bears a rounded pit anteriorly (Fig 9B2). The anterior face of the centrum is, to a large extent, concealed by the matrix except in the dorsal half where a part of the preceding centrum is preserved. The neural arches are in the form of low and narrow ridges and enclose a wide (2.5 cm) neural canal. The thick anterior and posterior edges enclosing a spindle-shaped depressed area in the mid-ventral face of the centrum are interpreted as the haemal arches.

There are three centra in the Kachchh ichthyosaur collection, which can be referred to the postflexural vertebrae (Figs 9A1–9A3 and 10A1–10C2). KGMV 0505, the best preserved of the three centra, has a width of 1.6 cm, height of 1.8 cm and length of 1.3 cm. The centra are small in size, elliptical in anterior and posterior views, biconcave, slightly compressed laterally and higher than transversely wide. Their dorsal surfaces are comparatively shorter than their ventral surfaces. The facets for neural arches are low, ridge-like, and transversely wide in the middle. There are no facets for ribs on these vertebrae. Similarly, no facets for haemal arches are present on the ventral face of the centra.

**Fig 10:**
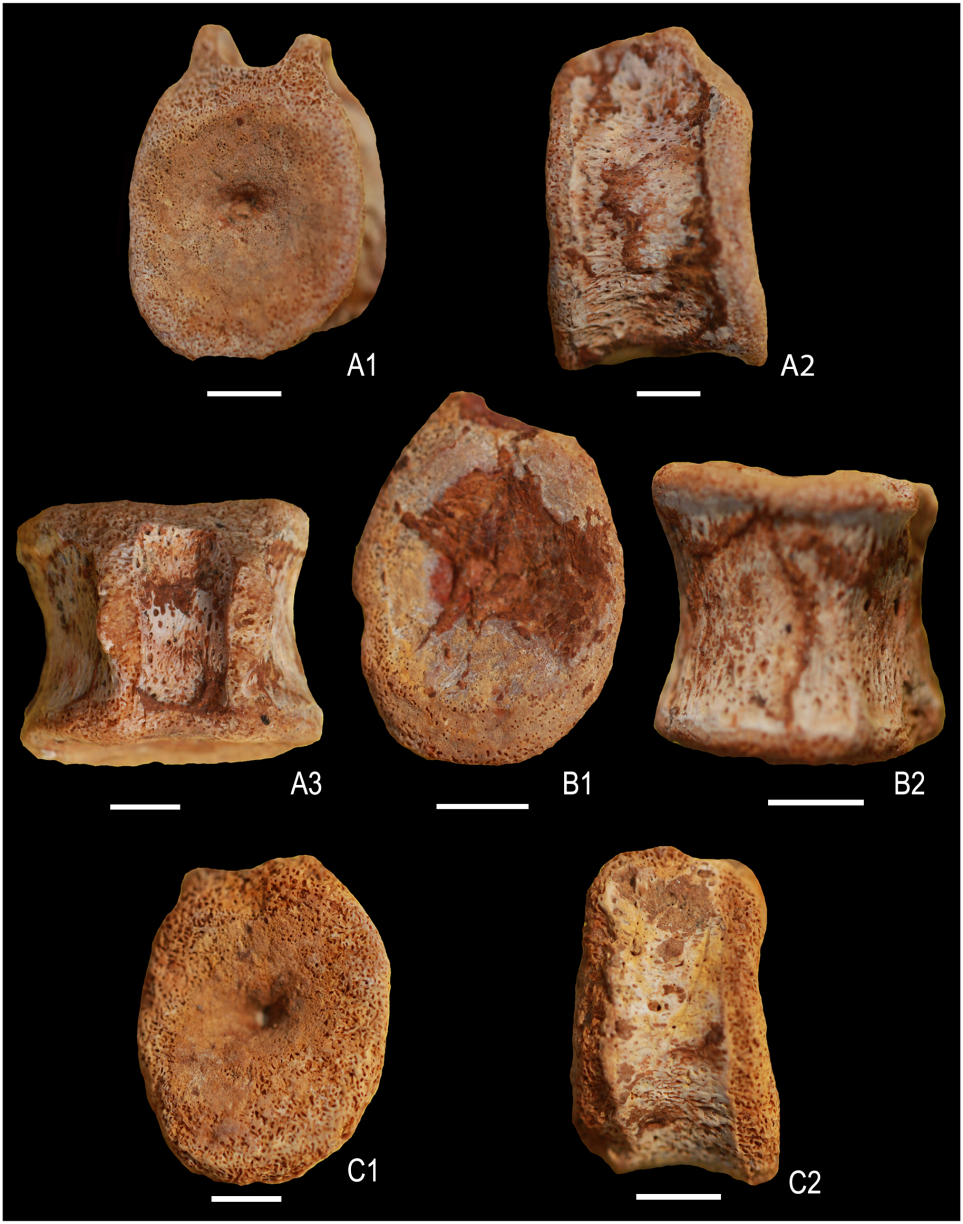
Post-flexural caudal vertebrae of Ophthalmosauridae gen. et sp. indet (A1-C2). A1-A2 (KGMV 0505), anterior or posterior view (A1), lateral view (A2), dorsal view (A3). B1-B2 (KGMV 0506), anterior or posterior view (B1), ventral view (B2). C1-C2 (KGMV 0507), anterior or posterior view (C1), lateral view (C2). Scale bars equal 1 cm.

#### Ribs

One proximal shaft of an isolated rib lying dorsally and near to the posterior end of the vertebral column is double-headed and has an elliptical cross-section (Fig 3A and 3B). The remaining ribs lie on the ventral side of the axial skeleton and are attached to the vertebrae, relatively thin, laterally compressed and highly fragmented. Many of the ribs occurring articulated in the anterior region are double- headed. The shafts of the ribs are flattened and grooved both anteriorly and posteriorly rendering a cross-sectional shape of ‘8’. Ribs near the presacral region are shorter, slender, and have elliptical to spherical cross-sections. In majority of the ribs, only proximal parts are preserved. The length of the longest preserved rib is 100 cm.

#### Neural spines

Except for the posterior region beyond the displaced? right forefin, the neural spines are preserved all along the dorsal surface of the vertebral column (Fig 3A and 3B). They are short, rectangular in shape with their long axis directed dorsally, decrease in size posteriorly, and occur well-separated from the vertebral centra.

#### Gastralia

A few clustered, short, rod-like bones of gastralia are found in the posterior region of the skeleton just anterodorsal to the preserved? right forefin (Fig 3A and 3B). They are cylindrical in outline and are also highly fractured as in the case of ribs. These bones are thinner than the ribs.

#### Left forefin

**Fig 11:**
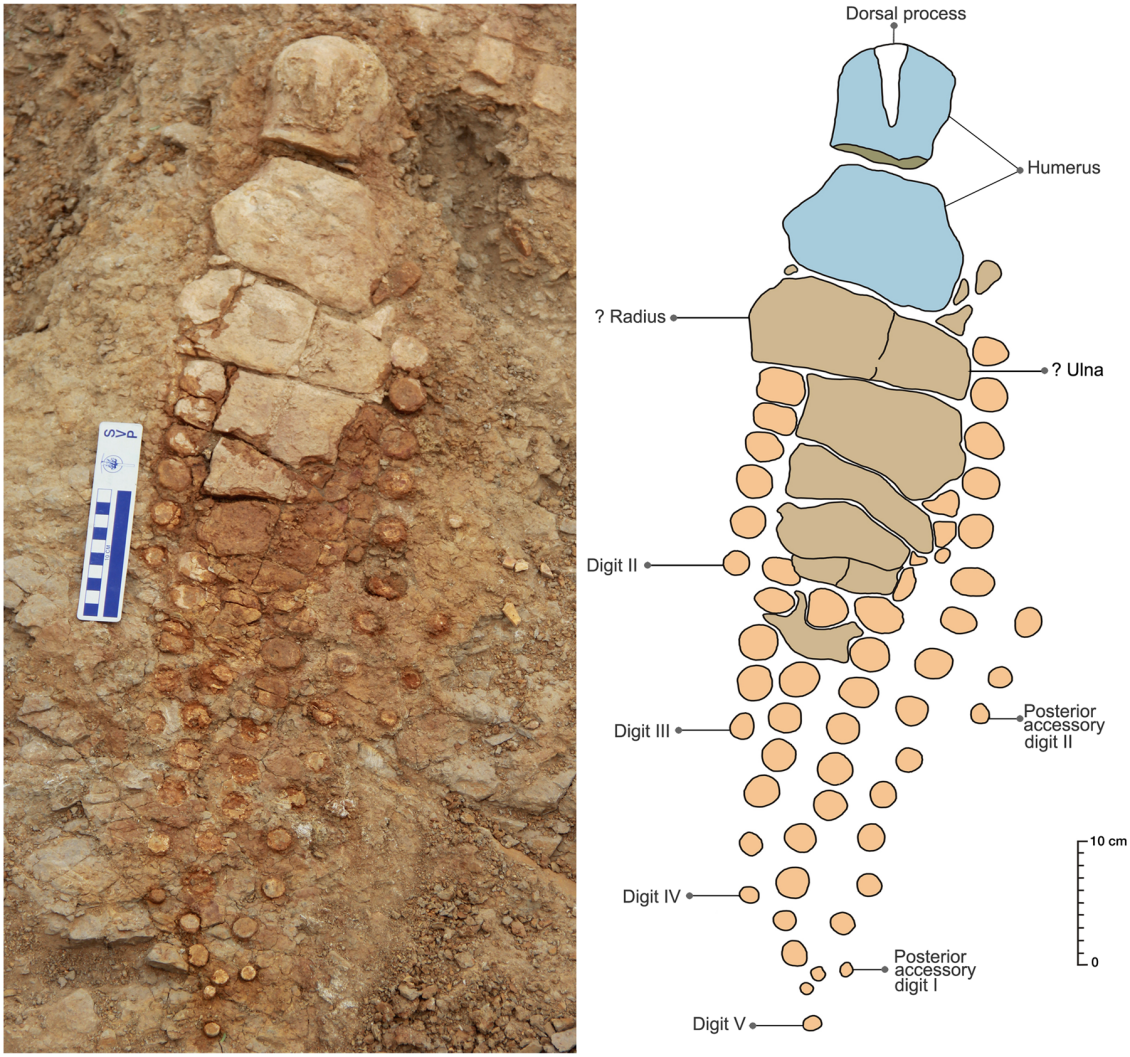
Close-up view of the left forefin accompanied with a sketch.

The exposed surface of the left forefin (Fig 11) is considered as dorsal as the humerus has a prominent dorsal process that extends distally one-third of the proximodistal length of the humerus. The dorsal process is positioned at equidistance from its anterior and posterior margins. The proximal extremity of the humerus is rounded in outline, while its distal end is moderately expanded anteroposteriorly. The anterior margin of the humeral shaft is moderately concave, whereas its posterior margin is slightly concave or nearly straight. The distal articular face is nearly straight except for a short, deflected facet anteriorly. There is a slight concavity in the middle of the distal face where the radius comes into contact with the humerus. The short obliquely oriented facet at its anterodistal end is possibly for articulation with the pre-radial element. Distal to the humerus, there are two large, rectangular elements, the anterior one being larger proximodistally as well as anteroposteriorly than the posterior one. Whether these elements represent the radius and ulna or it is the matrix that conceals these proximal fin elements will be clear only when the specimen is fully prepared. The sutures between the proximal elements of the forefin cannot be determined because of the poor contrast between the matrix and the bone. Post-depositional break-up of bones (longitudinal breaks) due to weathering further complicates their identification. Because of the concealment of the proximal part of the fin by the rock matrix, the proximal and distal carpals and metacarpals, which are crucial for generic identification, are not exposed. The fin is substantially long (80 cm) and slender (Table 1). Spherical disc-like phalanges are arranged in six rows. There appear to be four primary digits that begin distal to the metacarpals, and extend to the distal end of the fin. There are two accessory digits located posterior to the primary digits. The actual number of phalanges in the primary digits cannot be counted as their proximal ends are buried beneath the matrix. The anterior-most digit has only six exposed spherical elements that are located quite proximal to the other three primary digits. This could be a pre-axial digit or Digit II. The primary Digit III shows only five exposed phalanges and extends slightly distal to the Digit II. The fourth primary digit (Digit IV) has seven exposed phalanges and extends near to the distal end. The fifth primary digit (Digit V) extends to the distal-most part of the fin and has 12 exposed phalanges. The first posterior accessory digit is very long and starts from the posteroproximal end of the forefin. It has 15 spherical elements, which decrease in size distally. The second posterior accessory digit is located at the mid-length of the fin and has only three very small, disc-like phalanges. In the pre-axial digit or Digit II, the proximal two exposed phalanges appear to be rectangular in outline. It is, therefore, quite possible that the proximal most phalanges of the digits are rectangular or anteroposteriorly elongated. Majority of the exposed phalanges are clearly spherical in outline and gradually decrease in size distally. Though the phalanges are not closely packed in each digit, the individual digits are clearly differentiated from each other. The distal part of the forefin is posteriorly curved.

**Table 1:** Measurements of the humerus and left forefin. The measurements were taken from the proximal end of the humerus to the last phalanx including the break in the humerus and gaps between the phalanges.

	**Proximodistal length**	**Maximum anteroposterior width**	
**Left forefin**	80 cm	19 cm	
	**Proximodistal length**	**Width of proximal end**	**Width of distal end**
**Humerus**	25.5 cm	9.0 cm	14.5 cm

#### ?Right forefin

**Fig 12:**
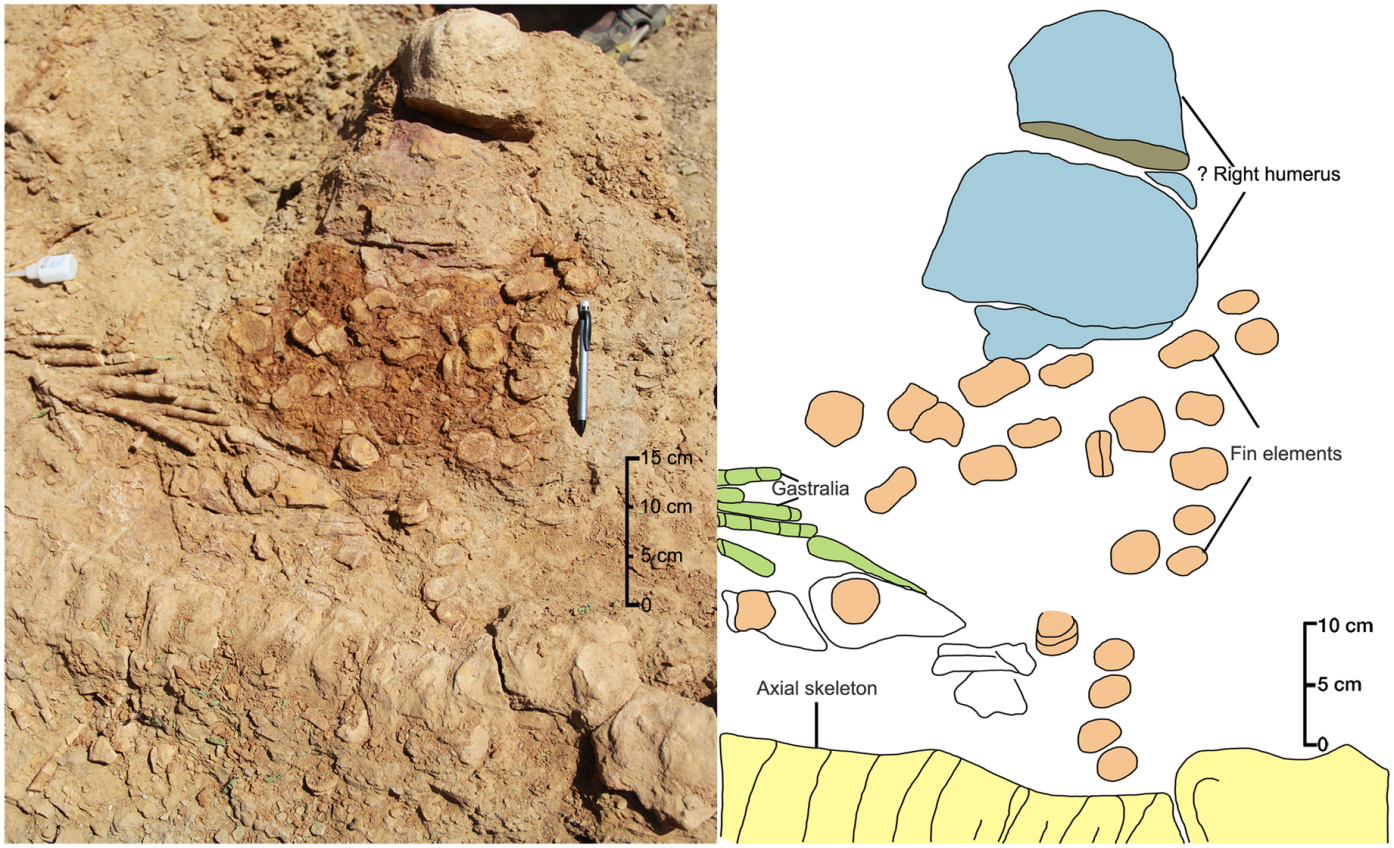
Close-up view of the ?right forefin accompanied with a sketch.

There is a second fin (Fig 12) located at the posterior end of the skeleton. The proximal bone of this fin is morphologically comparable to the humerus of the left forefin. Although the bone has a thick ferruginous coating concealing most of the morphological features, it appears to have a deltopectoral crest. This bone occurring at the posterior end of the articulated vertebral column has its distal extremity pointing towards the vertebral column rather than away from it. Therefore, it is inferred that this fin was displaced from its original position after the death of the animal. Its larger size is highly unusual for a pelvic fin because Late Jurassic ichthyosaurs had highly reduced hindfins [43]. The fin elements distal to the humerus are dislocated from their natural position and many of them are rectangular in shape. But half of the preserved phalanges, which are more distal in position and some located dorsoventral to the posterior presacral vertebrae, are ovoid or spherical in shape. The isolated rectangular phalanges recovered from the surface have a central groove all along the periphery of the bone. These rectangular elements may represent proximal elements of the fin.

#### Phalanges

Three isolated phalanges were surface-collected from the site of the articulated skeleton (KGMV 0508–0510). One of them is rectangular in outline (KGMV 508), dorsoventrally thick and most likely a proximal one (Fig 8B1 and 8B2). Two of them (KGMV 0509–0510) are comparatively small in size and disc-like (Fig 8C1 and 8C2) possibly representing the distal elements.

## Discussion

The Callovian and post-Callovian ichthyosaurs are generally included in the family Ophthalmosauridae [8, 9, 46–50], although Fischer et al. [6] reported a non-ophthalmosaurid ichthyosaur from the Early Cretaceous of Iraq. Keeping in view their chronostratigraphic position (Kimmeridgian Stage), the described specimens are referred to post-Liassic ophthalmosaurids. This conclusion further receives support from the presence of a stout dorsal process on the humerus of the forefin. This character has been viewed as a synapomorphy of Ophthalmosauridae in several phylogenetic analyses [8,10, 49, 51, 52]. The presence of an anterior accessory element articulating with the humerus was also considered as characteristic of ophthalmosaurids [11]. The amphicoelous isolated vertebrae from Kachchh are quite distinct from those of weakly amphicoelous vertebrae of platypterygiine ophthalmosaurids.

The documented fossil record of Middle-Late Jurassic ichthyosaurs is highly skewed towards the western Tethys (Anglo-Paris Basin), North America, and Northern Europe (Norway, Russia). But recent reports of ophthalmosaurid ichthyosaurs from Argentina, Mexico, Cuba, Madagascar, Australia and now from India clearly indicate that the apparent restricted distribution is due to poor sampling in these parts of the world. In their classic work, McGowan and Motani [3] considered five genera and 14 species of ophthalmosaurids as valid. Since then many new taxa have been reported from different parts of the world taking the list to 20 genera and 27 species [53]. Until now the Middle-Late Jurassic members of Ophthalmosauridae are known by 11 genera and 15 species (Table 2).

**Table 2:** Stratigraphic and geographic distribution of Middle and Late Jurassic ophthalmosaurid ichthyosaurs.

NAME OF THE TAXON	FORMATION & GEOGRAPHIC LOCATION	AGE
*Caypullisaurus bonapartei* [7]	Vaca Muerta Formation, Neuquén Basin, Argentina	Berriasian—Tithonian
*Palvennia hoybergeti* [11]	Agardhfjellet Formation, Svalbard, Norway	Tithonian
*Ophthalmosaurus* cf. *O*. *icenicus* [54]	La Caja Formation, Mexico	Tithonian
*Cryopterygius kristiansenae* [11]	Agardhfjellet Formation, Svalbard, Norway	Tithonian
*Cryopterygius kielanae* [55]	Kcynia Formation, Central Poland	Tithonian
*Undorosaurus gorodischensis* [56]	Volga Region and Moscow Region, Russia	Tithonian
*Undorosaurus trautscholdi* [57]	Mnevniki, Moscow, Russia	Tithonian
*Arthropterygius* sp. [9]	Vaca Muerta Formation, Neuquén Basin, Argentina	Tithonian
*Arthropterygius* sp. [13]	Paromes Formation, Russia	Middle Tithonian
*Janusaurus lundi* [12]	Agardhfjellet Formation, Svalbard, Norway	early Middle Tithonian
*Aegirosaurus leptospondylus* [58, 59]	Solnhofen Formation, Germany	Early Tithonian
*Nannopterygius enthekiodon* [60]	Kimmeridge Clay Formation, Dorset, England	Kimmeridgian
*Brachypterygius extremus* [61]	Kimmeridge Clay Formation, Dorset, England	Kimmeridgian
*Arthropterygius chrisorum* [10, 62]	Ringnes Formation, Melville Island, Northwest Territories, Canada	Kimmeridgian—Oxfordian
*Ophthalmosaurus natans* [63]	Sundance Formation, USA	Oxfordian—Callovian
*Ophthalmosaurus icenicus* [64]	Oxford Clay, England	Callovian
*Mollesaurus periallus* [15]	Los Molles Formation, Neuquén Basin, Argentina	Bajocian

**Fig 13:**
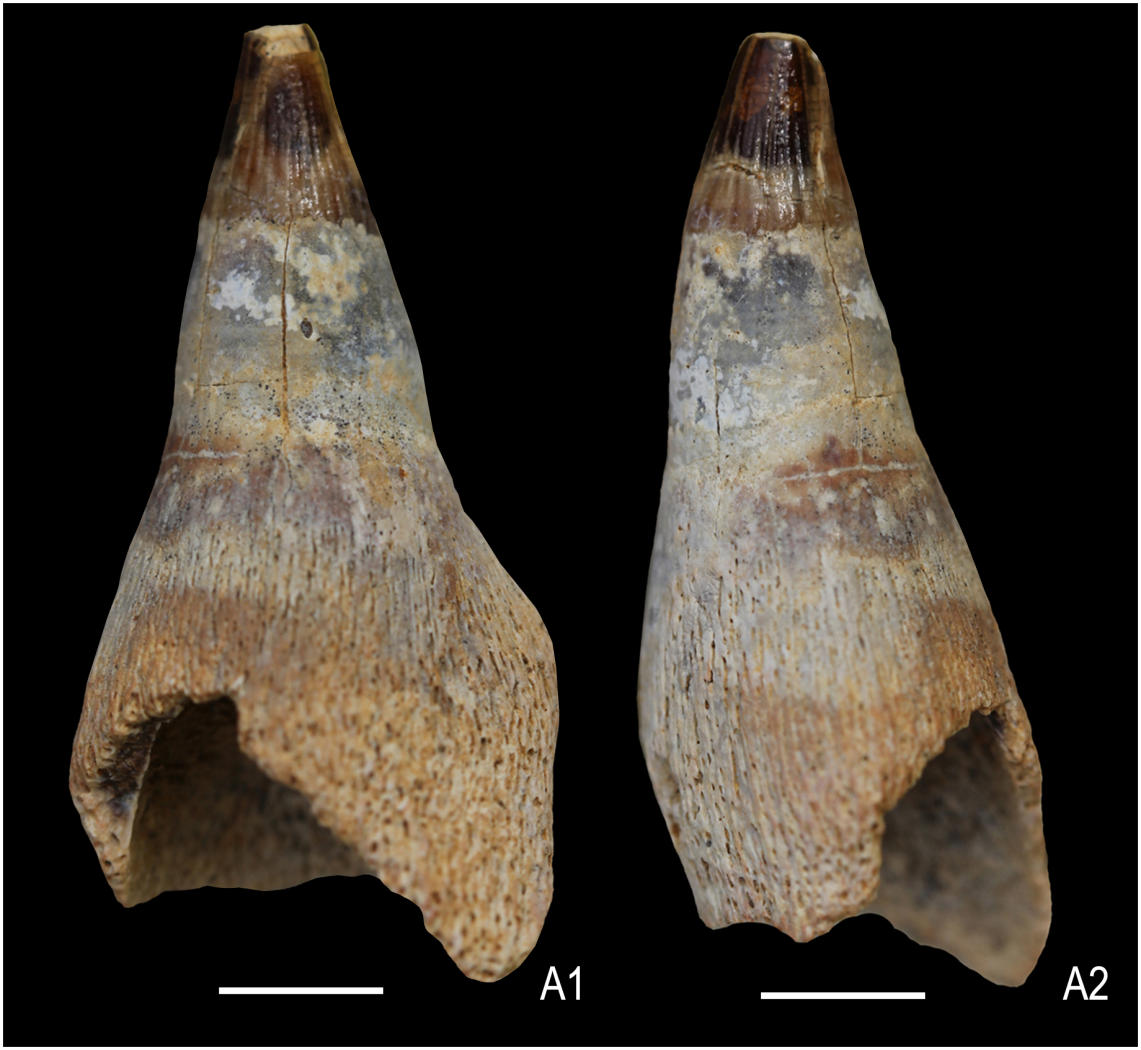
Isolated tooth of Platypterygiinae gen. indet. (DUGF/41) from the Upper Albian—Middle Cenomanian Karai Formation, Cauvery Basin, South India in lingual view (A1) and lateral view (A2). Scale bars equal 1 cm.

A squared root cross-section of *Platypterygius*, *Undorosaurus*, *Brachypterygius* and *Maiaspondylus* [56, 65, 66] was considered as a synapomorphy of platypterygiine ichthyosaurs [67]. A comparison of the ichthyosaur teeth from Kachchh is made with isolated ichthyosaur tooth (DUGF/41) from the Upper Albian—Middle Cenomanian Karai Formation of the Cauvery Basin, South India, assigned to Platypterygiinae gen. indet. [28]. The tooth of Platypterygiinae gen. indet. (Fig 13A1 and 13A2) is distinctly different from those of the studied specimens in having a comparatively short and less curved crown, and strongly basally expanded root with a rectangular cross-section. The Kachchh specimens with a rounded to oval root cross-section thus fall within the primitive ophthalmosaurine clade of the Ophthalmosauridae [67].

The little morphological information that could be deciphered from the proximal part of the left forefin distal to the humerus in its current state of preparation limits its comparison with other known ophthalmosaurids, but still some general conclusions can be made. In general, the outline of the forefin, being elongated and narrow anteroposteriorly, is comparable to that of *Aegirosaurus* from the Early Tithonian of Germany [59]. The humerus of *Aegirosaurus* is widest at the distal end as in the Kachchh specimen [59]. However, *Aegirosaurus* has a broad and distally less extended dorsal process situated at the proximal end of the humerus, an angulated distal margin, and more tightly packed fin elements which are to a large extent rectangular in outline. *Cryopterygius kristiansenae* from the Tithonian of Svalbard, Norway [11], has also a narrow and long fin. In *Cryopterygius*, however, the dorsal process of the humerus arises from the postaxial margin and its long axis trends anterodistally towards the midline and the fin is slightly asymmetrical.

In the morphology of the humerus, the Kachchh specimen differs from most members of the family Ophthalmosauridae in having a narrow proximal end narrower than a moderately broad distal end and a nearly straight distal end obliquely oriented to the long axis of the forefin as compared to the angulated facets separated by a ridge in the middle in most ophthalmosaurids. The humerus of the left forefin (KGMV 0501) with a well constricted mid-shaft and a distal end more anteroposteriorly expanded than the proximal end differs from that of *Arthropterygius*, *Undorosaurus*, *Cryopterygius*, *Janusaurus*, *Sveltonectes*, *Maiaspondylus* and *Platypterygius*. In *Caypullisaurus*, the distal end of the humerus is obliquely oriented to the long axis with a short angulation at the anterodistal end and the fin is long as in the Kachchh specimen ([7]: fig.5). However, the fin elements are rectangular and closely packed in *Caypullisaurus* [7]. In the Kachchh specimen, the distal humerus has three recognizable facets as in *O*. *icenicus*, *Brachypterygius*, *Arthropterygius*, *Caypullisaurus*, *Undorosaurus*, *Aegirosaurus*, *Janusaurus*, *Platypterygius* and *Acamptonectes* [7, 12, 45, 56, 59, 67] as compared to two facets in *Cryopterygius*, *Nannopterygius* and *Sveltonectes* [68]. In fact, the distal humerus of *Arthropterygius* compares well with that of the Kachchh specimen in possessing two relatively large facets for ulna and radius and a small, obliquely oriented preaxial facet [13]. But in *Arthropterygius*, a median crest separates the ulna from the radius.

The better-preserved left forefin of the Kachchh specimen has six digits as in *Cryopterygius*, *Aegirosaurus* and *Brachypterygius*. The phalanges of this specimen are rounded, widely spaced and dorsoventrally thick as in Ophthalmosauridae and their closest sister taxon *Chacaicosaurus* [68]. In many ophthalmosaurids, such as *Caypullisaurus*, *Platypterygius*, *Brachypterygius*, *Aegirosaurus* and *Maiaspondylus*, the fin elements are rectangular in outline and tightly packed [68]. In the wide spacing of elements (loose packing) and their rounded shape (particularly in the distal region), the left forefin of KGMV 0501 compares well with that of *Ophthalmosaurus icenicus* though the fin is broader in its middle, distal to the epipodials in the latter. The distal end of the humerus is also nearly straight in *O*. *icenicus* as in the Kachchh specimen though angulated posteriorly in the former. The relatively dorsoventrally thick elements of the fins are characteristic of the family Ophthalmosauridae [3]. However, the tentatively identified right forefin has dorsoventrally thick, rectangular phalanges proximally and rounded phalanges distally. The exposed phalanges in the left forefin are rounded in shape, while the shape of proximal elements appear to be rectangular but most of this area is concealed beneath the matrix. Thick proximal elements and the unnotched anterior facet of the elements on the leading edge of the fins are regarded as synapomorphies of Ophthalmosauridae and *Chacaicosaurus* [68].

In its shape, transverse width and height proportions, and location of the single rib facet slightly below the mid-height, KGMV 0504 compares very well with the one figured by Kirton ([45]: fig.20 h-j) as a posterior caudal vertebral centrum of *Ophthalmosaurus icenicus* from the Oxford Clay. However, McGowan and Motani [3] noted that isolated vertebrae, and their shape variation, are to be used taxonomically with caution.

### Dental wear and dietary preference

Besides using to distinguish between different groups of ichthyosaurs [69] and sometimes as an important taxonomic criterion [70], tooth shapes have been widely used in the past to deduce the preference of the animal for a particular prey or varied preys [69, 71]. Based on the shape of the tooth apex, wear pattern, presence or absence of cutting edges, as well as shape and size of the crown, Massare [71] defined seven feeding types or guilds for Mesozoic marine reptiles viz., crush guild, crunch guild, smash guild, pierce I guild, pierce II guild, general guild, and cut guild.

The two isolated teeth (KGMV 0502–0503) found on the surface close to the skeleton are embedded in the maroon-coloured ferruginous matrix and their surfaces were exposed during preparation. These teeth have finely fluted crowns with blunt conical apices and do not show any apical wear except for a rugose appearance and smoothening. KGMV 0502 displays a narrow strip (ribbon-like) of longitudinal wear on the enamel of the crown on its labiolateral face. Preliminary preparation of the premaxilla exposed one nearly complete large tooth (Fig 6A2). It has a worn (smooth), leveled tip (Fig 6A1), and a small spalled enamel on the labial face close to the apex (Fig 6A3). It also exhibits an oval or lenticular longitudinal spalled enamel surface extending from the apex to nearly the base of the crown on its lingual face (Fig 6A4). An interesting feature of this spalled wear facet of the enamel is the presence of two coarse longitudinal grooves that are oriented oblique to the wear surface (Fig 6A5). This kind of wear surfaces has not been reported in any of the known ichthyosaurs until now.

Dental wear surfaces due to attrition (tooth/tooth contact) or abrasion (tooth/food contact) on teeth are not very common in non-mammalian tetrapods [72]. While studying dental occlusion in *Dakosaurus*, a metriorhynchid crocodile from the Late Jurassic of Europe, Young et al. [72] noticed three types of wear: (1) apical wear or spalling of teeth or breakage of tip suggesting large prey or abrasive food that may include bone or both, and enamel spalling present on the labial or lingual surface of the tooth in the form of a discrete ovoid or triangular facet that begins at the apex of the crown and extends basally; (2) macroscopic wear along the mesial and distal carinae of the teeth that extends from the apex and terminates at variable distance from the base or occasionally extending along the whole length of the carinae; (3) semi-circular macroscopic wear present at the base of the crown and formed during the final phase of occlusion when the apex of the opposing tooth comes into contact with the base of the crown.

In case of the premaxillary tooth of the Kachchh specimen, the apex is not broken but worn flat. There is another in-situ tooth in the premaxilla that shows a broken crown apex (Fig 6B). It is, however, difficult to assess whether this was a post-mortem breakage or the result of abrasion with hard prey as a few other teeth in the premaxilla have broken tips. Enamel spalling is present on the labial face close to the apex. Similarly, a longitudinal ovoidal spalling of the enamel begins at the apex and extends very close to the base of the crown. The longitudinal wear strip found in KGMV 0502 may in fact represent the early stage of apical spalling. However, Young et al. [72] have not observed the obliquely oriented longitudinal grooves of the studied premaxillary tooth on spalled enamel surfaces of *Dakosaurus*. In contrast, the wear surfaces on the mesial and distal carinae and the semi-circular wear facet at the base of the crown characteristic of marine metriorhynchid crocodiles are not present on the premaxillary tooth of KGMV 0501. Examination of mosasaur, plesiosaur and ichthyosaur skeletons housed at the AMNH by Young et al. [72] revealed apical spalling in many instances as in the Kachchh specimen, but no wear on the mesial and distal carinae was noticed [72].

According to the definition of Massare [71], the teeth from Kachchh with an acute but rounded and polished apex are similar to the morphology of smash and crunch guilds. Enamel spalling is caused by the impact between the tooth and prey (an unusually hard substance such as bone) in the same axis in which the jaw closes [73], or by breaking the prey items into smaller pieces. The robustness of the teeth preserved in the premaxilla and extensive enamel spalling on one of the surfaces indicates that the teeth were used to grasp a prey with hard exterior such as armoured fish, crustaceans, and thick-shelled ammonites as in the case of the crunch guild of Massare [52, 71]. Robust teeth with intense tooth wear, frequent apical tooth breakage and enamel spalling are generally observed in top-tier predators [69]. Therefore, the wear pattern observed in the premaxillary tooth from Kachchh, which has not been earlier reported in ichthyosaurs, indicates that the animal was feeding on a very hard, abrasive prey and might have been a top-tier predator.

### Palaeobiogeographic significance

Middle and Late Jurassic ichthyosaurs primarily represented by ophthalmosaurids have been widely documented from the western Tethys (England, France and Germany), the Boreal region (Russia, Norway) and North America [3]. However, prospecting for Mesozoic vertebrate faunas in Gondwanan continents in recent years has corrected this bias towards Laurasian continents to some extent. Many discoveries of ichthyosaur fossils from Argentina [7, 9, 14, 15, 17], Chile [74], Antarctica [21, 22], Madagascar [19] and New Zealand [20] have improved the Gondwanan fossil record.

The Jurassic ichthyosaurs from South America (Argentina and Chile) are shown to be closely related to those from the Tethys region [18, 74]. Based on the common occurrence of the same genera and species of marine invertebrates in both regions [75–79], teleost fishes [80], herpetofauna [81], as well as marine reptiles [7, 54, 82] it has been suggested that the Caribbean or Hispanic Corridor connecting the eastern Pacific Ocean with the western Tethys through the Central Atlantic facilitated faunal dispersals between the two regions during the Late Jurassic [83–85].

However, a second dispersal route extending from Iraq-Kurdistan, East Africa, western India, Mozambique, Madagascar, and eastern Antarctica to the interconnected rift grabens (Rocas Verdes Basin) of southern Patagonia was also suggested based on the presence of similar bivalve, belemnite and ammonoid faunas in the Upper Jurassic rocks of these areas [76, 86]. The latter sea route has been designated as the Trans-Erythrean Seaway [87], Indo-Madagascan Seaway [88], South African Rocas Verdes Seaway [74], or Indo-Austral Seaway [85]. Currently, the timing of formation of this Trans-Gondwana Seaway is not well constrained. Though there is clear evidence for the prevalence of marine conditions in the early Middle Jurassic time from East Africa, Madagascar, and western India [89–94], Mozambique might have remained in contact with East Antarctica for a longer time, opening only in the Late Jurassic as evidenced by the formation of the first oceanic crust in the Mozambique Channel [95–97]. It has been proposed that this channel, which connected eastern Africa with the southern Andes along Antarctica, had been in existence since the Callovian, but became fully operational only from the Tithonian onwards [98]. Zverkov et al. [13] based on the common occurrence of *Arthropterygius* in Northern Europe, Argentina and Canada suggested that the southern seaway might have become operational by the Late Tithonian and Berriasian.

The Jurassic succession of the Kachchh Basin contains marine fauna that is very similar to that of Madagascar and therefore belongs to the Ethiopian or Indo-Madagascan faunal province. Based on the study of nautiloids, Halder [99] suggested that during the early stage of marine transgression Kachchh was an isolated basin promoting the evolution of endemic species. By the Late Bathonian, a two-way migration was possible between the Mediterranean Tethys and the Indo-Madagascan province. Finally, most species were common to both provinces in the Oxfordian and the Tethyan shallow shelf [100] was the primary migration route between these provinces.

In the Jurassic fauna of Kachchh, there are several examples of episodic faunal exchanges between the Indo-Madagascan and the Mediterranean provinces. These include the Callovian reineckeiids [101], *Indosphinctes*, Oxfordian *Larcheria* [102], Kimmeridgian *Nebrodites* and Late Kimmeridgian—Early Tithonian *Hybonoticeras* [103]. The appearance of ammonid genera characteristic of different palaeobiogeographic provinces, such as *Tithopeltoceras* and *Durangites* of the Mediterranean Province and *Himalayites* of the Mediterranean Tethys and Indo-Madagascan Province in the Late Tithonian fauna of Kachchh has been explained in terms of interprovincial migrations facilitated by high sea level [104–107]. Many of the ostracod taxa reported from the Callovian-Oxfordian Chari Formation and lower part of the Kimmeridgian Katrol Formation also demonstrate close affinities to those of Rajasthan (western India), Madagascar, Tanzania and central Saudi Arabia thus favouring faunal exchanges between the western Tethys and the Indo-Madagascan Province as early as in Oxfordian-Kimmeridgian time [108].

A two-way marine dispersal route between Kachchh and the Andes Basin via Madagascar and South Africa has also been visualized in view of the occurrence of *Corongoceras* cf. *C*. *lotenoense* in the Tithonian of Kachchh and *Virgatosphinctes*, *Blanfordiceras*, *Spiticeras* in the Andes [76, 106]. Similarly, *Megacucullaea*, a bivalve, considered to be endemic to the Indo-Madagascan Province, reached the Andes in the Late Jurassic [76, 104, 107].

**Fig 14:**
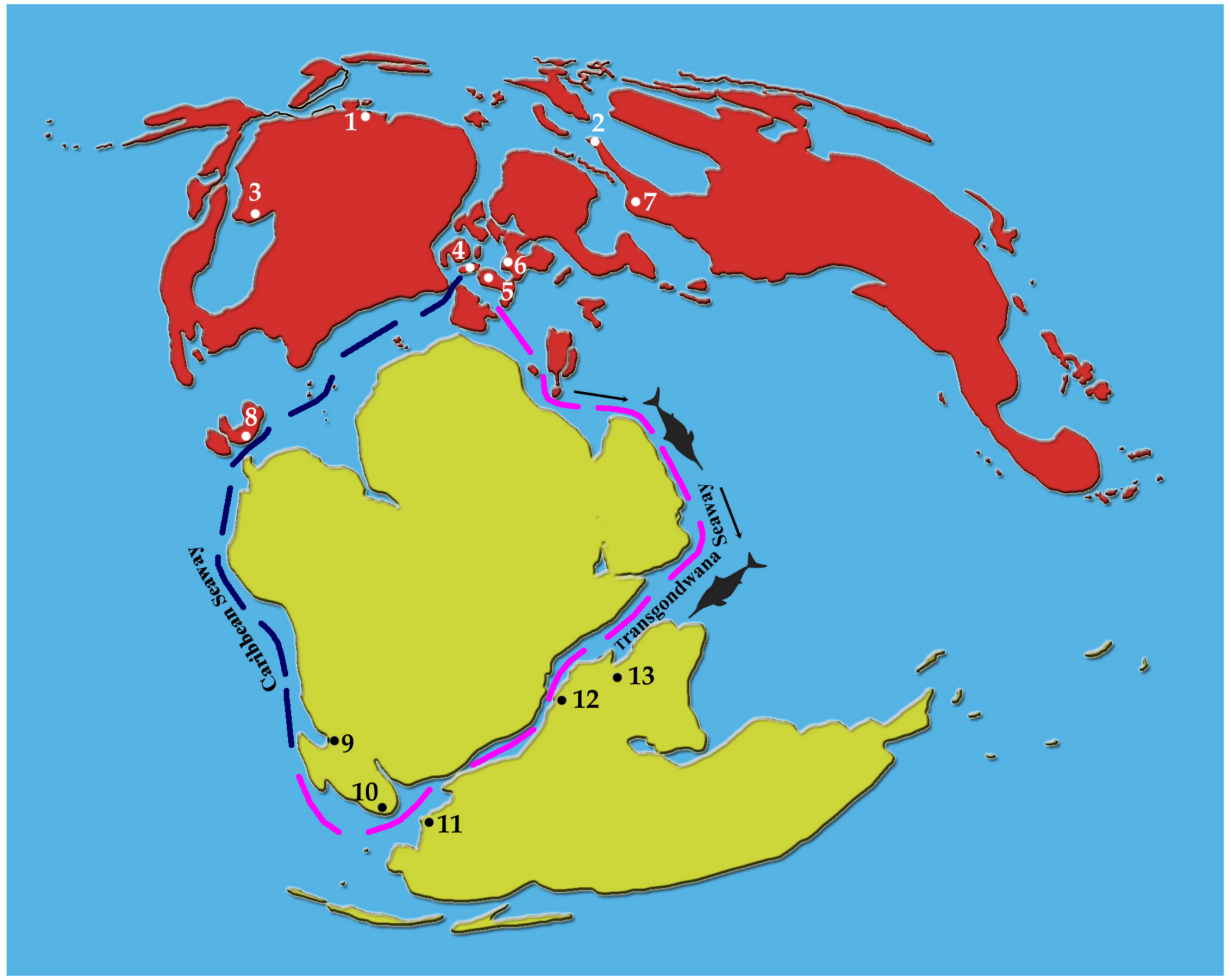
Late Jurassic world palaeogeographic map showing the distribution of Middle and Late Jurassic ophthalmosaurids (adopted from fig 3 of Fernández [19]). 1. Canada (*Arthropterygius*), 2. Norway (*Cryopterygius*, *Brachypterygius*, *Palvennia*, *Janusaurus*), 3. USA (*Ophthalmosaurus*), 4. England (*Ophthalmosaurus*, *Brachypterygius*, *Nannopterygius*), 5. Germany (*Aegirosaurus*), 6. Poland (*Cryopterygius*), 7. Russia (*Ophthalmosaurus*, *Brachypterygius*, *Arthropterygius*, *Undorosaurus*), 8. Mexico (*Ophthalmosaurus*, *Brachypterygius*), 9. Argentina (*Ophthalmosaurus*, *Caypullisaurus*, *Arthropterygius*), 10. Chile (Ophthalmosauridae indet.), 11. Antarctica (Ichthyosauria *incertae sedis*), 12. Madagascar (Ophthalmosauridae indet.), 13. India (Ophthalmosauridae indet.). The inferred sea route along which faunal interchanges may have taken place between the western Tethys and the southern Indian Ocean is shown in magenta colour.

Prior to the present ichthyosaur discovery, a cryptoclidid plesiosaur, a Middle-Late Jurassic plesiosaur widely known from western Europe, was documented from the Upper Jurassic Katrol Formation [30]. This marine reptile also supports the existence of faunal interchanges between the Indo-Madagascan and western Tethys provinces in the Late Jurassic (Kimmeridgian). Based on the temperature minimum recorded from the early Late Oxfordian of Kachchh, it was suggested that the widening of the Trans-Gondwanan Seaway may have led to increased upwelling in the Malagasy Gulf and to a cooling recorded in the oxygen isotopes of belemnites and other marine invertebrates from Kachchh [38]. While describing an ophthalmosaurid ichthyosaur forefin and basioccipital from the Tithonian of Madagascar, Fernández [19] also indicated that this marine reptile find might support the southern Trans-Gondwana route. The present report of a partially preserved skeleton belonging to an ophthalmosaurid ichthyosaur underscores the fact that ichthyosaurs had a wide geographic distribution as previously suggested [109]. Further, the presence of ophthalmosaurid ichthyosaurs in the Upper Jurassic strata of western India, Madagascar, Argentina and Chile with close affinities to those of the western Tethys in addition to the reported presence of ichthyosaurs in the Jurassic of Antarctica [21, 22] supports the possible existence of a Trans-Gondwana dispersal route (Fig 14) as early as in the Kimmeridgian as suggested earlier on the basis of invertebrate fossils. Though the circuitous trans-Pacific migration route along the Caribbean Seaway for the dispersal of ophthalmosaurids from the western Tethys to Indo-Madagascan province is not impossible, the Trans-Gondwana route is the most parsimonious one in light of additional existing evidence from bivalves, cephalopods and plesiosaurs. A more clear picture on the southern biogeographic connections will emerge as and when the phylogenetic relationships of KGMV-0501 with the Upper Jurassic ophthalmosaurids is precisely established.

## Conclusions

The present ichthyosaur find represents the first nearly complete articulated skeleton of an ichthyosaur from India and the first record from the Jurassic of India. It further expands our knowledge on morphological diversity and geographic distribution of Late Jurassic ophthalmosaurids, their dietary habits and palaeobiogeography. At present, the specimen cannot be positively identified with known Jurassic ichthyosaur taxa from other parts of the world because of its encrustation in ferruginous rock matrix, which conceals much of the morphological information. In its dental characters and vertebral and forefin morphology, KGMV 0501 is referable to the ichthyosaur family Ophthalmosauridae. However, the currently available limited number of characters is inadequate to establish generic or species level relationships within the family. In general, the morphology of humerus and forefin indicate close similarities with *Ophthalmosaurus*, *Arthropterygius* and *Aegirosaurus*.

The preserved axial skeleton measures 3.6 m in length. If the partially preserved snout (36 cm), the missing posterior skull region and post-flexural tail are taken into account, the ichthyosaur from Kachchh may have had an estimated length of 5.0–5.5 m. In this respect, it may represent an adult animal that was comparatively large in size with respect to other Late Jurassic ichthyosaurs [7, 59]. Its state of preservation indicates that the animal landed on the sea floor vertically on its snout, while the rest of the body later fell on its side. The body has also been subjected to limited displacement and disarticulation following its deposition on the sediment surface possibly due to scavenging or reworking. The wear pattern on the teeth suggests that the animal was adapted for feeding on a hard and abrasive prey.

Although the taxonomic position of the Kachchh ichthyosaur within the family Ophthalmosauridae is unresolved at the moment, it represents one of the few ichthyosaur finds from the Jurassic of the least explored former Gondwanaland. It adds to the already known ichthyosaur discoveries of Argentina, Madagascar, New Zealand, Australia, Antarctica and Chile thereby improving the fossil record of this group of marine reptiles from the former Gondwanaland. The presence of ophthalmosaurid ichthyosaurs in the Upper Jurassic of India, Madagascar and South America implies that a marine seaway possibly connected the western Tethys with South America via the Indian Ocean in the Late Jurassic facilitating faunal exchanges between Europe and Gondwanan continents. Identification of the Kachchh specimen below family level is crucial for understanding its phylogenetic relationships to contemporaneous taxa from the former Gondwanaland and Laurasia and consequent palaeobiogeographic connections. The articulated nature of the Kachchh ichthyosaur and the previous record of plesiosaur remains from the Kimmeridgian and Tithonian rocks of the Kachchh Mainland further indicates that focused research in remote areas of Kachchh may lead to more exciting finds in the future.

## Abbreviations

AMNH: American Museum of Natural History
DUGF/: Delhi University, Department of Geology fossil collection numbers
KGMV: Kachchh Geological Museum Vertebrate Fossil catalogue numbers
RUC2016IKH: Rajasthan University 2016 collection from Kas Hill section

## References

[1] Fischer V, Bardet N, Benson RBJ, Arkangelsky MS, Friedman M. Extinction of fish-shaped marine reptiles associated with reduced evolutionary rates and global environmental volatility. Nature Communications. 2016; 7:10825/ doi: 10.1038/ncomms10825/www.nature.com/naturecommunications. 26953824 10.1038/ncomms10825 PMC4786747

[2] Ji C, Jiang D-Y, Motani R, Rieppel O, Hao W-C, Sun Z-Y. Phylogeny of the Ichthyopterygia incorporating recent discoveries from South China. Journal of Vertebrate Paleontology. 2015.

[3] McGowan C, Motani R. Part 8. Ichthyopterygia In: Sues H-D, editor. Handbook of Paleoherpetology. München: Verlag Dr. Friedrich Pfeil; 2003 pp. 1–175.

[4] Thorne PM, Ruta M, Benton MJ. Resetting the evolution of marine reptiles at the Triassic-Jurassic boundary. PNAS. 2011; 20:8339–8344. 10.1073/pnas.1018959108 PMC3100925 21536898

[5] Fernández MS. Ophthalmosauria (Ichthyosauria) forefin from the Aalenian-Bajocian boundary of Mendoza province, Argentina. Journal of Vertebrate Paleontology. 2003; 23:691–694.

[6] Fischer V, Appleby RM, Naish D, Liston J, Riding JB, Brindley S, et al A basal thunnosaurian from Iraq reveals disparate phylogenetic origins for Cretaceous ichthysaurs. Biology Letters. 2013; 9: 20130021 http://dx.doi.org/10.1098/rsbl.2013.0021. 23676653 10.1098/rsbl.2013.0021 PMC3730615

[7] Fernández MS. A new ichthyosaur from the Tithonian (Late Jurassic) of the Neuquén Basin, northwestern Patagonia, Argentina. Journal of Paleontology. 1997a; 71:479–484.

[8] Fernández MS. Redescription and phylogenetic position of Caypullisaurus (Ichthyosauria: Ophthalmosauridae). Journal of Paleontology. 2007; 81:677–681.

[9] Fernández MS, Maxwell EE. The genus *Arthropterygius* Maxwell (Icthyosauria: Ophthalmosauridae) in the Late Jurassic of the Neuquén Basin, Argentina. Geobios. 2012; 45:535–540.

[10] Maxwell EE. Generic reassignment of an ichthyosaur from the Queen Elizabeth Islands, Northwest Territories, Canada. Journal of Vertebrate Paleontology. 2010; 30:403–415.

[11] Druckenmiller PS, Hurum JH, Knutsen EM, Nakrem HA. Two new ophthalmosaurids (Reptilia: Ichthyosauria) from the Agardhfjellet Formation (Late Jurassic: Volgian/Tithonian), Svalbard, Norway. Norwegian Journal of Geology. 2012; 92:311–339.

[12] Roberts AJ, Druckenmiller PS, Saetre GP, Hurum JH. A new Upper Jurassic ophthalmosaurid ichthyosaur from the Slottsmøya Member, Agardhfjellet Formation of Central Spitsbergen. PLoS ONE. 2014; 9(8): e103152 doi: 10.1371/journal.pone.0103152 25084533 10.1371/journal.pone.0103152 PMC4118863

[13] Zverkov NG, Arkhangelsky MS, Pardo-Pérez JM, Beznosov PA. On the Upper Jurassic ichthyosaur remains from the Russian North. Proceedings of the Zoological Institute RAS. 2015; 319(1):558–588.

[14] Fernández MS. A new long snouted ichthyosaur from the early Bajocian of Neuquén Basin (Argentina). Ameghiniana. 1994; 31:291–297.

[15] Fernández MS. A new ichthyosaur from the Los Molles Formation (Early Bajocian), Neuquén basin, Argentina. Journal of Paleontology. 1999; 73:677–681.

[16] Fernández MS. Late Jurassic ichthyosaurs from the Neuquén Basin, Argentina. Historical Biology. 2000; 14:133–136.

[17] Fernández MS, Talevi M. Ophthalmosaurian (Ichthyosauria) records from the Aalenian—Bajocian of Patagonia (Argentina): an overview. Geological Magazine. 2014; 151:49–59.

[18] Pardo-Peréz J, Frey E, Stinnesbeck W, Fernández MS, Rivas L, Salazar C, et al An ichthyosaurian forefin from the Lower Cretaceous Zapata Formation of southern Chile: Implications for morphological variability within *Platypterygius*. Palaeobiodiversity and Palaeoenvironments. 2012; 92:287–294.

[19] Fernández MS. On the paleogeographical distribution of the Callovian and Late Jurassic ichthyosaurs. Journal of Vertebrate Paleontology. 1997b; 17: 752–754.

[20] Zammit M. Australasia’s first Jurassic ichthyosaur fossil: an isolated vertebra from the Lower Jurassic Arataura Formation of North Island, New Zealand. Alcheringa. 2011; 35(3):341–343.

[21] Whitham AG, Doyle P. Stratigraphy of the Upper Jurassic Lower Cretaceous Nordenskjöld Formation of eastern Graham Land, Antarctica. Journal of South American Earth Sciences. 1989; 2(4):371–384.

[22] Hikuroa DCH. Second Jurassic marine reptile from the Antarctic Peninsula. Antarctic Science. 2009; 21(2):169–170.

[23] Lydekker R. Fossil Reptilia and Batrichia. Palaeontological Indica Series.Palaeontologica Indica Series. 1879; IV(1): 1–36.

[24] Lydekker R. Note on the classification of the Ichthyopterygia with a notice of two new species. Geological Magazine 3rd Series. 1888; 5:309–314.

[25] Underwood CJ. Goswami A, Prasad GVR, Verma O, Flynn JJ. Marine vertebrates from the ‘mid’ Cretaceous (early Cenomanian) of South India. Journal of Vertebrate Paleontology. 2011; 31:539–552.

[26] Ayyasami K, Elamparuthi S, Gowtham B. An ichthyosaur vertebra from the Cretaceous (Middle Cenomanian) Karai Formation, southern India. Journal Geological Society of India. 2016; 87:706–708.

[27] Zammit M. Cretaceous ichthyosaurs: Dwindling diversity, or the empire strikes back? Geosciences. 2012; 2:11–24.

[28] Fischer V. Taxonomy of Platypterygius campylodon and the diversity of the last ichthyosaurs. PeerJ; 2016 4:e2604 doi: 10.7717/peerj.2604 27781178 10.7717/peerj.2604 PMC5075704

[29] Verma KK, Satsangi PP, Srivastava S, Mehra S. On a plesiosaurian vertebra from the upper Gondwanas of Kutch, Gujarat, India. In: Laskar B, Raja Rao CS, editors. Fourth International Gondwana Symposium, Proceedings and Papers (India 1977). Calcutta: 1979. pp. 217–220.

[30] Bardet N, Mazin JM, Cariou E, Enay R, Krishna J. Les Plesiosauria du Jurassique supérieur de la province de Kachchh (Inde). Comptes Rendus de l’Académie des Sciences de Paris 313, 1991; Série.II:1343–1347.

[31] Lydekker R. Notices of new and other Vertebrata from Indian Tertiary and Secondary rocks. Records of the Geological Survey of India. 1877; 10:41.

[32] Phansalkar VG, Sudha G, Kadkikar AS. Giant marine reptilian skulls from the Jurassic of Kachchh, Gujarat. Current Science. 1994; 67(6): 460–461.

[33] Biswas SK. Stratigraphy and sedimentary evolution of the Mesozoic basin of Kutch, Western India In: Tandon SK, Pant CC, Casshyap SM, editors. Stratigraphy and sedimentary evolution of western India. Nainital: Gyanodaya Publications; 1991 pp.74–103.

[34] Wynne AB. Memoir of the geology of Kutch to accompany the map compiled by A.B. Wynne and F. Fedden during the seasons of 1867–68 and 1868–69. Memoirs of the Geological Survey of India. 1872; 9(1):1–293.

[35] Waagen W. The Jurassic fauna of Cutch. Memoirs of the Geological Survey of India, Palaeontologia Indica. 1873–1875; 9:1–247.

[36] Fürsich FT, Singh IB, Joachimski M, Krumm S, Schlirf M, Schlirf S. Palaeoclimate reconstructions of the Middle Jurassic of Kachchh (western India): an integrated approach based on palaeoecological, oxygen isotopic, and clay mineralogical data. Palaeogeography, Palaeoclimatology, Palaeoecology. 2005; 217:289–309.

[37] Fürsich FT. Environmental distribution of trace fossils in the Jurassic of Kachchh (western India). Facies. 1998; 39:243–272.

[38] Alberti M, Fürsich FT, Pandey DK. The Oxfordian isotope record (δ^18^O, δ^13^C) of belemnites, brachiopods, and oysters from the Kachchh Basin (western India) and its potential for palaeoecologic, palaeoclimatic and palaeogeographic reconstructions. Palaeogeography, Palaeoclimatology, Palaeoecology. 2012; 344–345:49–68.

[39] Fürsich FT, Alberti M, Pandey DK. Stratigraphy and palaeoenvironments of the Jurassic rocks of Kachchh—Field Guide. Beringeria. 2013; 7:1–174. 28.

[40] Krishna J, Pathak DB, Pandey B. Quantum refinement in the Kimmeridgian ammonoid chronology in Kachchh (India). GeoResearch Forum. 1996; 1–2: 195–204.

[41] Krishna J, Pandey B, Ojha JR. *Gregoryceras* in the Oxfordian of Kachchh (India): diverse eventful implications. Geobios. 2009; 42:197–208.

[42] Martill DM. Soupy substrates: a medium for the exceptional preservation of ichthyosaurs of the Posidonia Shale (Lower Jurassic) of Germany. Kaupia. 1993; 2:77–97.

[43] Delsett LL, Roberts AJ, Druckenmiller PS, Hurum JH. A new ophthalmosaurid (Ichthyosauria) from Svalbard, Norway, and evolution of the ichthyopterygian pelvic girdle. PLoS ONE. 2017; 12(1): e0169971 doi: 10.1371/journal.pone.0169971 28121995 10.1371/journal.pone.0169971 PMC5266267

[44] Sollas WJ. The skull of *Ichthyosaurus* studied in serial sections. Philosophical Transactions of the Royal Society of London. 1916; Series B 208: 62–126.

[45] Kirton AM. A review of British Upper Jurassic ichthyosaurs. Ph.D. Thesis, University of Newcastle upon Tyne. 1983. 239 p. http://ethos.bl.uk/OrderDetails.do?uin=uk.bl.ethos.344855.

[46] Motani R. On the evolution and homology of ichthyosaurian forefins. Journal of Vertebrate Paleontology. 1999a; 19:28–41.

[47] Maisch MW, Matzke AT. The Ichthyosauria. Stuttgarter Beiträge zur Naturkunde, Serie B. 2000; 298:1–159.

[48] Sander PM. Ichthyosauria: their diversity, distribution, and phylogeny. Paläontologische Zeitschrift. 2000; 74:1–35.

[49] Druckenmiller PS, Maxwell EE. A new lower Cretaceous (lower Albian) ichthyosaur genus from the Clearwater Formation, Alberta, Canada. Canadian Journal of Earth Sciences. 2010; 47:1037–1053.

[50] Maisch MW. Phylogeny, systematics, and origin of the Ichthyosauria—the state of the art. Palaeodiversity. 2010; 3:151–214. 66.

[51] Motani R. Phylogeny of the Ichthyopterygia. Journal of Vertebrate Paleontology. 1999b; 19: 473–496.

[52] Fischer V, Clément A, Guiomar M, Godefroit P. The first definite record of a Valanginian ichthyosaur and its implication for the evolution of post-Liassic Ichthyosauria. Cretaceous Research. 2011b; 32:155–163.

[53] Fernández MS, Campos L. Ophthalmosaurids (Ichthyosauria: Thunnosauiria): Alpha taxonomy, clades and names. In: Fernández M, Herrera Y, editors. Reptiles Extinctos—Volumen en Homenaje a Zulma Gasparini. Publicacion Electronica de la Asociacion Paleontologica Argentina. 2015; 15(1):20–30.

[54] Buchy MC. First record of *Ophthalmosaurus* (Reptilian: Ichthyosauria) from the Tithonian (Upper Jurassic) of Mexico. Journal of Paleontology. 2010; 84(1):149–155.

[55] Tyborowski D. A new ophthalmosaurid ichthyosaur from the Late Jurassic of Owadów-Brzezinki Quarry, Poland. Acta Palaeontologica Polonica. 2016; 61(4): 791–803

[56] Efimov VM. A new family of ichthyosaurs, the Undorosauridae fam. nov. from the Volgian stage of the European part of Russia. Paleontological Journal. 1999; 33(2):174–181.

[57] Arkhangelsky MS, Zverkov NG. On a new ichthyosaur of the genus *Undorosaurus*. Proceedings of the Zoological Institute. 2014; 318(3): 187–196.

[58] Wagner A. Beschreibung einer fossilen Schildkröte und etlicher anderer Reptilien-Ueberreste aus den lithographischen Schiefern und dem Gründsandsteine von Kelheim. Abhandlungen der Mathematische-Physikalischen Classe der königlich bayerischen Akademie der Wissenschaften. 1853; 7(1):239–264.

[59] Bardet N, Fernández MS. A new ichthyosaur from the Upper Jurassic Lithographic Limestones of Bavaria. Journal of Paleontology. 2000; 74(3): 503–511.

[60] Hulke JW. Note on an Ichthyosaurus (I. enthekiodon) from Kimmeridge Bay, Dorset. Quarterly Journal of the Geological Society of London. 1871; 27:440–441.

[61] Boulenger GA. On a new species of ichthyosaur from Bath. Proceedings of the Zoological Society of London. 1904; 1904(1):424–426.

[62] Russell DA. Jurassic marine reptiles from Cape Grassy, Melville Island, Arctic Canada. The Geology of Melville Island, Arctic Canada. Geological Survey of Canada Bulletin. 1993; 450:195–201.

[63] Marsh OC. A new order of extinct reptiles (Sauranodonta) from the Jurassic Formation of the Rocky Mountains. American Journal of Science. 1879; Series 3(17):85–86.

[64] Seeley HG. On the pectoral arch and forelimb of *Ophthalmosaurus*, a new ichthyosaurian genus from the Oxford Clay. Quartely Journal of the Geological Society of London. 1874; 30:699–707.

[65] Bardet N. Dental cross-sections in Cretaceous Ichthyopterygia: Systematic implications. Geobios. 1990; 23:169–172.

[66] Maxwell EE, Caldwell MW. Evidence for a second species of the ichthyosaur Platypterygius in North America: a new record from the Loon River Formation (Lower Cretaceous) of northwestern Canada. Canadian Journal of Earth Sciences. 2006; 43:1291–1295.

[67] Fischer V, Maisch MW, Naish D, Liston J, Kosma R, Joger U, et al New ophthalmosaurid ichthyosaurs from the Early Cretaceous of Europe demonstrate extensive ichthyosaur survival across the Jurassic—Cretaceous boundary. PLoS ONE. 2012; 7(1):e29234 doi: 10.1371/journal.pone.0029234 22235274 10.1371/journal.pone.0029234 PMC3250416

[68] Fischer V, Masure E, Arkhangelsky MS, Godefroit P. A new Barremian (Early Cretaceous) ichthyosaur from western Russia. Journal of Vertebrate Paleontology. 2011a; 31:1010–1025.

[69] Fischer V, Bardet N, Guiomar M, Godefroit P. High diversity in Cretaceous ichthyosaurs from Europe prior to their extinction. PLoS ONE. 2014; 9(1):e84709 doi: 10.1371/journal.pone.0084709 24465427 10.1371/journal.pone.0084709 PMC3897400

[70] Lomax DR. A new leptonectid ichthyosaur from the Lower Jurassic (Hettangian) of Nottinghamshire, England, UK, and the taxonomic usefulness of the ichthyosaurian coracoid. Journal of Systematic Palaeontology. 2016; http://dx.doi.org/10.1080/14772019.2016.1183149.

[71] Massare JA. Tooth morphology and prey preference of Mesozoic marine reptiles. Journal of Vertebrate Paleontology. 1987; 7:121–137.

[72] Young MT, Brusatte SL, Beatty BL, Andrade MB, Desojo JB. Tooth-on-tooth interlocking occlusion suggests macrophagy in the Mesozoic marine crocodylomorph *Dakosaurus*. The Anatomical Records. 2012; 295:1147–1158. 10.1002/ar.22491 22577071

[73] Schubert BW, Ungar PS. Wear facets and enamel spalling in tyrannosaurid dinosaurs. Acta Palaeontologica Polonica. 2005; 50:93–99.

[74] Shultz MR, Fildani A, Suarez M. Occurrence of the Southernmost South American Ichthyosaur (Middle Jurassic—Lower Cretaceous), Parque Nacional Torres del Paine, Patagonia, Southernmost Chile. Palaios. 2003; 18:67–73.

[75] Hallam A. Early and Mid-Jurassic molluscan biogeography and the establishment of the central Atlantic seaway. Palaeogeography, Palaeoclimatology, Palaeoecology. 1983; 43:181–193.

[76] Riccardi AC. Jurassic and Cretaceous marine connections between the Southeast Pacific and Tethys. Palaeogeography, Palaeoclimatology, Palaeoecology. 1991; 87:155–189.

[77] Ballent SC, Whatley R. The distribution of the Mesozoic ostracod genus Procytherura Whatley, palaeogeographical implications with special reference to Argentina. Alcheringa. 2000; 24:229–242.

[78] Aberhan M. Bivalve palaeobiogeography and the Hispanic Corridor: time of opening and the effectiveness of a proto-Atlantic seaway. Palaeogeography, Palaeoclimatology, Palaeoecology. 2001; 165:375–394.

[79] Damborenea SE. Hispanic Corridor: Its evolution and the biogeography of bivalve molluscs. GeoResearch Forum. 2000; 6:369–380.

[80] Arratia G. The Jurassic and the early history of the teleosts In: Arratia G, Viohl G, editors. Mesozoic Fishes—Systematics and Paleoecology. München: Verlag Dr. Friedrich Pfeil; 1996 pp. 243–259.

[81] Gasparini Z. Marine Reptiles of the circum-Pacific region In: Westermann G, editor. The Jurassic of the Circum-Pacific. Cambridge: Cambridge University Press, World and Regional Geological Series; 1992 pp. 361–364.

[82] Fernández MS, Iturrade-Vinent M. An Oxfordian Ichthyosauria (Reptilia) from Viñales, Western Cuba: Paleobiogeographic significance. Journal of Vertebrate Paleontology. 2000; 20(1):191–193.

[83] Gasparini Z. A new Oxfordian pliosaurid (Plesiosauria, Pliosauridae) in the Caribbean Seaway. Palaeontology. 2009; 52:661–669.

[84] Gasparini Z, Fernández M, de la Fuente M, Salgado L. Reptiles marinos jurásicos y cretácicos de la Patagonia argentina: Su aporte al conocimiento de la herpetofauna mesozoica. Asociación Paleontológica Argentina Publicación Especial. 2007; 11:125–136.

[85] Salazar C. The Jurassic-Cretaceous boundary (Tithonian—Hauterivian) in the Andean Basin of central Chile: Ammonites, bio- and sequence stratigraphy and palaeobiogeography. Heidelberg: Ruprecht-Karls Universität; 2012 388 p.

[86] Leanza HA. The Tithonian ammonite genus Chigaroceras Howarth (1992) as a bioevent marker between Iraq and Argentina. GeoResearch Forum. 1996; 1–2:451–458.

[87] Arkell WJ. Jurassic Geology of the World. Edinburgh: Oliver and Boyd, 1956 806 p.

[88] Cecca F. Palaeobiogeography of Tethyan ammonites during the Tithonian (latest Jurassic). Palaeogeography, Palaeoclimatology, Palaeoecology. 1999; 147:1–37.

[89] Pandey DK, Fürsich FT. Bajocian (Middle Jurassic) Age of the Lower Jaisalmer Formation of Rajasthan, western India. Newsletters on Stratigraphy. 1994; 30:75–81. 36.38. 39.41.

[90] Förster R. The geological history of the sedimentary basin of southern Mozambique, and some aspects of the origin of the Mozambique Channel. Palaeogeography, Palaeoclimatology, Palaeoecology. 1975; 17:267–287.

[91] Singh CSP, Jaitly AK, Pandey DK. First report of some Bajocian—Bathonian (Middle Jurassic) ammonoids and the age of oldest sediments from Kachchh, W. India. Newsletters on Stratigraphy. 1982; 11(1):37–40.

[92] Krishna J. An overview of the Mesozoic stratigraphy of Kachchh and Jaisalmer basins. Journal of the Palaeontological Society of India. 1987; 32:136–49.

[93] Ali Kassim M, Carmignani L, Conti P, Fantozzi PL. Geology of the Mesozoic—Tertiary sedimentary basins in southwestern Somalia. Journal of African Earth Sciences. 2002; 34:3–20.

[94] Geiger M, Clark DN, Mette W. Reappraisal of the timing of the breakup of Gondwana based on sedimentological and seismic evidence from the Morondava Basin, Madagascar. Journal of African Earth Sciences; 2004; 38:363–381.

[95] Rabinowitz PD, Coffin MF, Falvey D. The separation of Madagascar and Africa. Science. 1983; 220:67–69. doi: 10.1126/science.220.4592.67 17736163 10.1126/science.220.4592.67

[96] Veevers JJ. Gondwanaland from 650–500 Ma assembly through 320 Ma merger in Pangea to 185–100 Ma breakup: supercontinental tectonics via stratigraphy and radiometric dating. Earth-Science Reviews. 2004; 68:1–132.

[97] Martin AK. Gondwana break-up via double-saloon-door rifting and seafloor spreading in a backarc basin during subduction rollback. Tectonophysics. 2007; 445:245–272.

[98] Enay R. Paleobiogeographie des Ammonites des Jurassique Terminal (Tithonique/Volgien/Portlandien S.L.) et Mobilite Continentale. Geobios. 1972; 5(4): 355–407.

[99] Halder K. Diversity and biogeographic distribution of Jurassic nautiloids of Kutch, India, during the fragmentation of Gondwana. Journal of African Earth Sciences. 2000; 311:175–185.

[100] Cairou E. Ammonites of the Callovian and Oxfordian In: Hallam A, editor. Atlas of Palaeobiogeography. Amsterdam: Elsevier; 1973 pp. 287–295.

[101] Cairou E, Krishna J. The Tethyan Reineckeiinae of Kachchh and Jaisalmer (West India): Systematic biostratigraphic and biogeographic implications. Palaeontographica A. 1988; 203:149–170.

[102] Krishna J, Melendez G, Pandey B, Pathak DB. Characterization of the ammonite genus Larcheria (Middle Oxfordian) in Kachchh (India): Paleontology, biostratigraphic and paleobiogeographic evaluation in context of north Tethyan occurrences. Comptes Rendus de l’Académie des Sciences de Paris. 1995; 321(2a):1187–1193.

[103] Krishna J, Pathak DB. Late Lower Kimmeridgian—Lower Tithonian virgatosphinctins of India: Evolutionary succession and biogeographic implications. Geobios. 1993; 15:227–238.

[104] Shome S, De S, Roy P, Bardhan S, Das SS. Ammonites as biological stopwatch and biogeographical blackbox—a case study from the Jurassic-Cretaceous boundary (150 Ma) of Kutch, Gujarat. Current Science. 2004; 86(1):197–202.

[105] Shome S, Bardhan S, De S. Record of *Tithopeltoceras* (Ammonoidea) from the Tithonian of Kutch, India and its stratigraphic and paleobiogeographic significance. Journal of Paleontology. 2005; 79(3): 619–624.

[106] Bardhan S, Shome S, Roy P. Biogeography of Kutch ammonites during the Latest Jurassic (Tithonian) and a global paleobiogeographic overview In: Landman NH, Davis RA, Mapes RH, editors. Cephalopod Present and Past: New Insights and Perspectives. Dordrecht: Springer; 2007 pp. 375–395.

[107] Shome S, Bardhan S. A new Late Tithonian ammonite assemblage from Kutch, Western India. Journal of the Palaeontological Society of India. 2009; 54(1):1–18.

[108] Khosla SC, Jakhar SR, Mohammed MH. Ostracodes from the Jurassic beds of Habo Hill, Kachchh, Gujarat, India. Micropaleontology. 1997; 43(1):1–39.

[109] McGowan C. Further evidence for the wide geographical distribution of ichthyosaur taxa (Reptilia: Ichthyosauria). Journal of Paleontology. 1978; 52:1155–1162.

